# F-box DNA Helicase 1 (FBH1) Contributes to the Destabilization of DNA Damage Repair Machinery in Human Cancers

**DOI:** 10.3390/cancers15184439

**Published:** 2023-09-06

**Authors:** Alizhah J. Watson, Michaela L. Shaffer, Renee A. Bouley, Ruben C. Petreaca

**Affiliations:** 1 Biology Program, The Ohio State University, Marion, OH 433023, USA; watson.1151@buckeyemail.osu.edu (A.J.W.); writesel.11@buckeyemail.osu.edu (M.L.S.); 2 Department of Chemistry and Biochemistry, The Ohio State University, Marion, OH 43302, USA; 3 Department of Molecular Genetics, The Ohio State University, Marion, OH 43302, USA; 4 Cancer Biology Program, James Comprehensive Cancer Center, The Ohio State University, Columbus, OH 43210, USA

**Keywords:** DNA damage, genetic recombination, helicase, cancer

## Abstract

**Simple Summary:**

Cancer cells are characterized by the accumulation of genetic mutations due to failures of the repair machinery. In some cases, excessive “repair” may cause DNA damage if the machinery runs amok and acts on DNA sequences that have not been damaged. In this report, we interrogate mutations in the FBH1 gene, which restrains homologous recombination, one mechanism of DNA double-strand break repair in eukaryotes. We find that mutations in FBH1 co-occur with mutations in the breast cancer susceptibility gene BRCA2 and other DNA damage repair genes. These findings suggest that FBH1 contributes to the general destabilization of the repair machinery in cancer cells.

**Abstract:**

Homologous recombination (HR) is the major mechanism of rescue of stalled replication forks or repair of DNA double-strand breaks (DSBs) during S phase or mitosis. In human cells, HR is facilitated by the BRCA2-BRCA1-PALB2 module, which loads the RAD51 recombinase onto a resected single-stranded DNA end to initiate repair. Although the process is essential for error-free repair, unrestrained HR can cause chromosomal rearrangements and genome instability. F-box DNA Helicase 1 (FBH1) antagonizes the role of BRCA2-BRCA1-PALB2 to restrict hyper-recombination and prevent genome instability. Here, we analyzed reported FBH1 mutations in cancer cells using the Catalogue of Somatic Mutations in Cancers (COSMIC) to understand how they interact with the BRCA2-BRCA1-PALB2. Consistent with previous results from yeast, we find that FBH1 mutations co-occur with BRCA2 mutations and to some degree BRCA1 and PALB2. We also describe some co-occurring mutations with RAD52, the accessory RAD51 loader and facilitator of single-strand annealing, which is independent of RAD51. In silico modeling was used to investigate the role of key FBH1 mutations on protein function, and a Q650K mutation was found to destabilize the protein structure. Taken together, this work highlights how mutations in several DNA damage repair genes contribute to cellular transformation and immortalization.

## 1. Introduction

Genetic recombination evolved first in bacteria to facilitate the replication of long genomes [1]. The process is so essential for replication that both the function and in some cases the structure of the enzymes has been preserved in eukaryotes [2,3]. The major function of recombination is to rescue stalled replication forks and repair DNA double-strand breaks (DSBs), which often arise during S phase as a consequence of replication stress [4,5,6,7]. This mechanism uses a homologous sequence to copy the missing or damaged information and is referred to as *H*omologous *R*ecombination (HR) [8]. In eukaryotes, other mechanisms, such as *N*on-*H*omologous *E*nd-*J*oining (NHEJ) and related pathways have also evolved to repair DSBs [9,10,11]. Although NHEJ can function throughout the cell cycle, its role increases in the nonreplicating cell stages especially in higher eukaryotes, such as humans. In sexually reproducing eukaryotes, recombination has also been co-opted for meiosis [12].

In both bacteria and eukaryotes, genome instability increases when recombination genes are mutated [13,14,15]. Cancer cells that often display high levels of genome instability are replete with mutations in HR and other DNA damage repair genes [16,17,18,19]. In human cells, HR is facilitated primarily by the BRCA2-BRCA1-PALB2 module, which loads RAD51 onto a resected single-stranded end to initiate a homologous search and strand exchange [20,21]. RAD52 has an assistant and parallel role with BRCA2 in human cells but is singularly required for loading RAD51 in yeast [22,23,24,25,26,27]. Several other accessory factors have been identified with various roles [28,29,30]. A plethora of nucleases and helicases are required to prepare broken ends, assist the RAD51 recombinase, resolve recombinant structures, and, in some cases, restrain recombination activities [31,32]. F-box helicase 1 (FBH1) was identified in yeast as a suppressor of RAD52 deletion. Its function is to limit the action of the RAD51 recombinase by counteracting the activity of RAD52 especially at stalled replication forks [33,34]. This HR suppressive function of FBH1 has been conserved in human cells [35,36] where FBH1 ubiquitylates RAD51 and restricts its recombination activities [37,38]. At replication forks, FBH1 also facilitates activation of the DNA damage checkpoint [39], restricts extensive resection and processing of fork intermediates [40], promotes repair of single-strand breaks arising from base alkylation [41], and suppresses extensive template switching, which causes chromosomal rearrangements [34]. FBH1 is recruited to replication forks via interaction with PCNA [42,43]. Thus, FBH1 is critical for ensuring accurate fork restart.

As expected, FBH1 mutations have been detected in cancer cells (Supplementary Table S1), but a comprehensive analysis of these mutations has not yet been carried out. Interestingly, a recent classification of driver genes in cancer cells [44] did not include FBH1 suggesting that changes in its activity (either loss- or gain-of-function) does not significantly impact cellular transformation and immortalization. Nevertheless, FBH1 mutations are expected to contribute to the overall mutation burden in cancer cells. In this report, we used artificial intelligence algorithms to characterize mutations, gene expression changes, and copy-number variations appearing in cancer cells with the goal to map the FBH1 mutation spectrum.

## 2. Materials and Methods

The FBH1 mutation file was downloaded from COSMIC version 97 (https://cancer.sanger.ac.uk/cosmic, accessed on 23 February 2023) as an Excel file. The data were processed with OpenCRAVAT to extract mutation impact probabilities. The data are publicly available on COSMIC but are attached as supplemental tables (Supplementary Tables S1 and S2).

**Figure 1:**
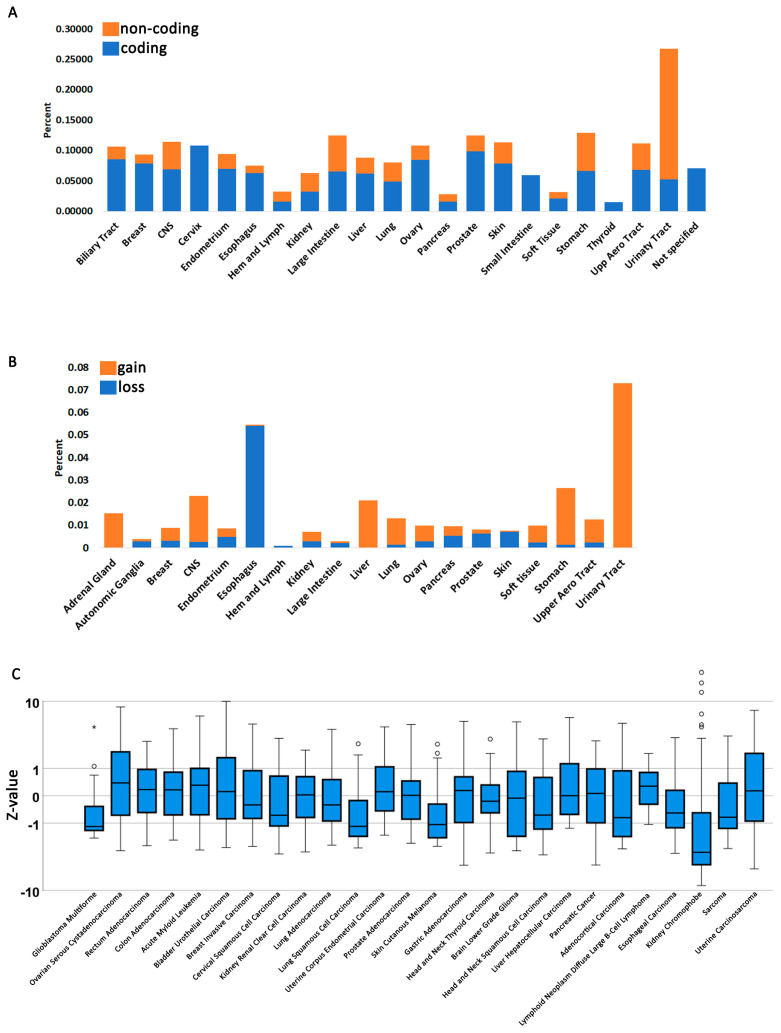
FBH1 mutation and expression profiles in human cancers. (**A**) FBH1 mutation percentage in all reported tissues. Shown are both coding (translated region) and noncoding (5′UTR, 3′UTR, and introns). (**B**) FBH1 copy-number gains or losses in cancer tissues reported on COSMIC. For both (**B**,**C**), the data are normalized to total mutation or CNV appearing in cancer cells. (**C**) FBH1 RNA transcript expression profiles for TCGA studies. The “*” symbol represents truncations.

For data in Figure 1A,B, FBH1 mutation and CNV counts were normalized to total aberrations appearing in cancer cells. To do this, the “COSMIC Mutation Data” or “Copy- Number Variants” whole files, which represent aberrations in all cancers, were downloaded and partitioned by cancer type. The FBH1 counts were then divided by total counts in each tissue and multiplied by 100 to compute percent mutation. For copy-number variants, loss or gain was taken as below or above 2.7 (e.g., +/−2.7) average ploidy as defined in [45].

The OpenCRAVAT (https://opencravat.org/, accessed on 23 February 2023) CHASMPlus artificial intelligence tool [46,47,48] was used to calculate the probability of FBH1 mutation impact on cancer.

Graphs and statistical analysis were performed in SPSS or Excel. All figures were made in Photoshop.

PyMOL was used to visualize the AlphaFold model of FBH1 and remove residues 1–215. The Q650K mutation was made using the PyMOL mutagenesis tool, and side-chain interactions were identified using the polar interaction tool. Graphs and statistical analysis were performed in SPSS or Excel. All figures were made in Photoshop.

## 3. Results and Discussion

### 3.1. Fbh1 Gene Alterations and Expression Profiles in Human Cancers

We queried all FBH1 mutations on COSMIC, which reports both TCGA studies as well as other independent studies described in the literature (Supplementary Table S1). Both coding (translated) and noncoding (5′ and 3′ UTRs and intronic) mutations were detected in nearly every cancer type (Figure 1A). We also investigated gene copy-number changes (Figure 1B, Supplementary Table S2). Gene copy-number alterations can occur due to changes in ploidy, such as gain or loss of entire chromosomes or intrachromosomal deletions or duplications. When data were normalized as a percent of total mutations occurring in each cancer type, we found that the esophagus is characterized by a hither percent of FBH1 loss, while the urinary tract shows more noncoding mutations than other tissues and has generally FBH1 copy-number gain. To our knowledge, this has not been previously reported. Finally, we characterized FBH1 gene expression levels (Figure 1C, Supplementary Table S2). COSMIC reports gene expression levels for certain TCGA studies obtained either from microarray analysis or RNA seq as a Z-value with values above Z = 2 considered overexpressed and under Z = −2 under-expressed. A value between −2 and 2 is interpreted as normal expression [49,50]. Several samples show a tendency toward under-expression, but the Kidney Chromophobe study is the most pronounced.

### 3.2. FBH1 Coding Mutations

**Figure 2:**
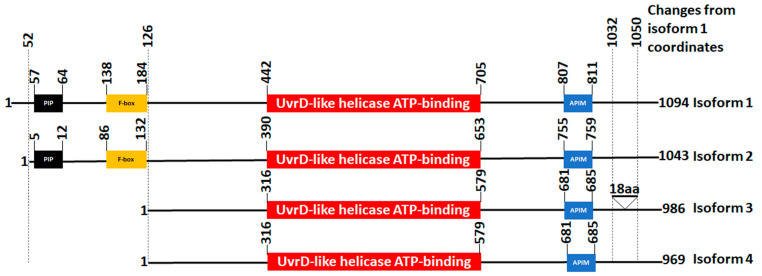
FBH1 isoforms. Four different isoforms are reported. Please see text for details.

Coding mutations are more likely to affect protein function than noncoding mutations because they change the sequence of the peptide. We therefore investigated all the FBH1 coding mutations in human cancers. FBH1 was first identified in *S. pombe* [51] and then subsequently in humans [52]. The helicase contains the canonical F-box motif at its N-terminus and the catalytic helicase domain in the center of the protein (Figure 2). At least four FBH1 protein isoforms resulting from alternate transcripts have been identified in humans. Unfortunately, COSMIC reports mutations using isoform 1 coordinates, but AlphaFold and CHASM artificial intelligence algorithms report mutations on isoform 2 causing some confusion. For example, the same mutation when reported on isoform 2 coordinates will be shifted by 51 amino acids if reported on isoform 1. We provide a diagram with all four isoforms aligned for easier interpretation of mutations discussed here (Figure 2). This diagram should also facilitate a more seamless interpretation of mutation coordinates reported by various databases for the four isoforms.

Both isoforms 1 and 2 have a PCNA-interacting peptide (PIP) domain at their N-terminus. The PIP domain is required for PCNA-dependent degradation of FBH1 via the Cullin ring ligase 4, an E3 ubiquitin ligase [43]. Isoform 2 is missing a 51 amino acid sequence at its N-terminus before the PIP domain. Isoforms 3 and 4 are lacking a significant part of their N-termini, which includes both the PIP and F-box domains. All isoforms contain an AlkB homolog 2 PCNA-interacting motif (APIM) at their C-termini. Biochemical and structural analyses show that APIM binds the same PCNA site as PIP, but PIP has a higher affinity for PCNA than APIM [43]. Isoform 3 also contains an additional 18-amino-acid sequence at its C-terminus.

**Figure 3:**
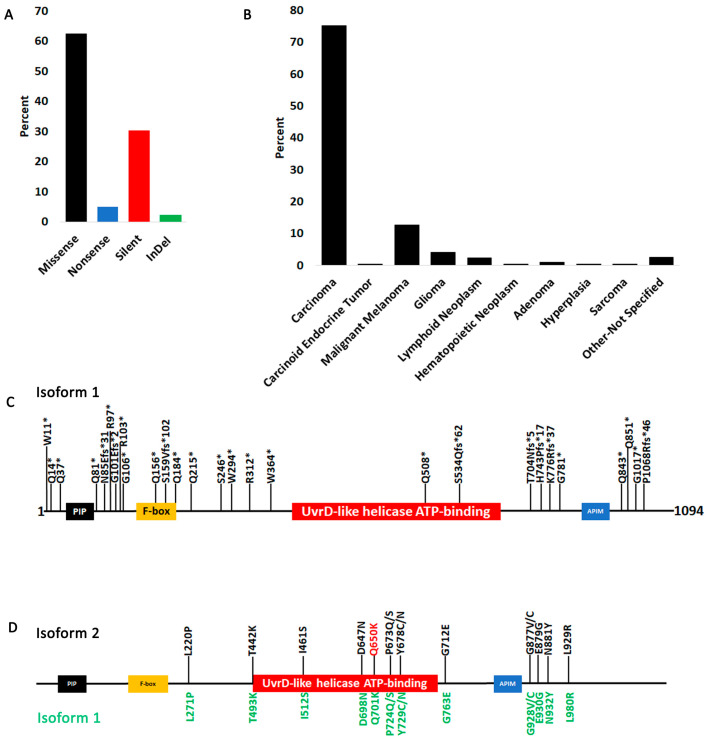
Most significant FBH1 mutations. (**A**) Type of FBH1 coding mutations. (**B**) Distribution of FBH1-coding mutations by cancer histology. (**C**) A map of all FBH1-truncating mutations on isoform 1. The “*” next to the amino acid represents position of truncation (e.g., in Q81* the truncation occurs at amino acid 81). If the mutation is an InDel and introduces a frameshift, the “*” indicates the position where the truncation will occur (e.g., in N86Efs*31, a frameshift occurs at amino acid 86 which would introduce a stop codon and truncation 31 amino acids downstream from residue 86). (**D**) A map of FBH1 point mutations with a CHASMPlus *p*-value below 0.1 on isoform 2 with corresponding isoform 1 coordinates below. The Q650K mutation has a *p*-value of 0.0471.

Most FBH1 mutations are missense and silent though some nonsense (truncating), and InDel were also identified (Figure 3A). Most cancer histology types queried in this COSMIC analysis were carcinomas (Figure 3B). We identified quite a few truncating mutations, many of them in the C-terminus of the protein that eliminate the helicase-active site of the protein (Figure 3C). COSMIC provides data on mutation zygosity for only 13.1% of the coding mutations (75 out of 572). All truncating mutations except E900* for which data are provided are heterozygous. To classify the point mutations that are not truncating, we used the CHASM artificial intelligence algorithm [48] using the OpenCRAVAT website [53], which computes the probability of a mutation being a driver. The algorithm is first trained on TCGA data using previously characterized driver and passenger mutations, then it could be used to assess the status of new mutations reported by non-TCGA studies. As expected, given that FBH1 is not classified as a driver gene, all mutations except one do not have a statistically significant *p*-value (Supplementary Table S2), suggesting that they do not substantially contribute to cellular transformation and immortalization. Figure 3D shows only those mutations with a *p*-value of 0.1 or below. Only Q650K has a *p*-value below 0.05 (*p* = 0.0471). OpenCRAVAT outputs isoform 2 coordinates, and AlphaFold also only has an isoform 2 structure; therefore, the coordinates in Figure 3D are diagramed on isoform 2 with corresponding coordinates on isoform 1 shown in green below.

### 3.3. Fbh1 Co-Occurring Mutations with Other Recombination Genes

**Figure 4:**
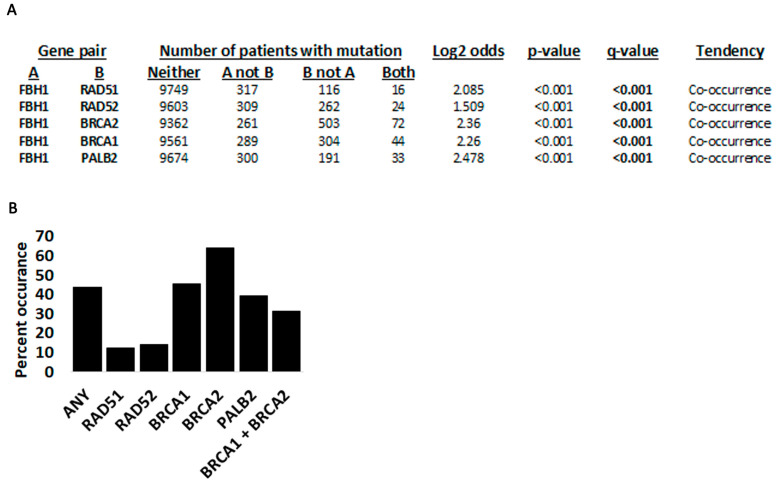
Co-occurring mutations between FBH1 and RAD51, RAD52, BRCA1, BRCA2, and PALB2. (**A**) Probability of co-occurring mutations between the indicated genes extracted from cBioPortal. Shown are computational probabilities between any gene pair. For example, for the FBH1 and RAD51 pair, 9749 samples have mutations in neither gene, 317 have a mutation in FBH1 but not RAD51, 116 have a mutation in RAD51 but not FBH1, and 16 have a mutation in both genes. The algorithm calculates Log2 odds as well as *p-* and q-values. (**B**) Percent of FBH1-co-occurring mutations with the genes indicated using data from COSMIC. Summary of Supplementary Table S3.

We next checked for FBH1 co-occurring mutations with RAD51, as well as the BRCA2-BRCA1-PALB2 module and RAD52, which are required for loading RAD51 (Figure 4, Table 1, Supplementary Table S3). cBioPortal predicts a tendency of co-occurring mutations between FBH1 and any of the other five genes for the TCGA pan-cancer studies (Figure 4A). Using the COSMIC data, we found that mutations between FBH1 and BRCA2 were most frequent, while those between FBH1 and BRCA1 or FBH1 and PALB2 were second- and third-most frequent, respectively. FBH1 mutations rarely co-occur with RAD52, the backup RAD51 loading enzyme, even when the BRCA2-BRCA1-PALB2 axis is functional (Supplementary Table S3, Table 1, Figure 4B). Although this analysis may suggest that RAD52-FBH1 co-mutations may not be as tolerated in cancer cells, this interpretation has a major caveat. RAD52 is only 418 amino acids, while BRCA2 is 3418 amino acids, BRCA1 is 1863 amino acids, and PALB2 is 1186 amino acids [54]. Because we checked for any mutations between these genes regardless of their impact on the function of the protein, statistically speaking, it is more likely to find mutations between longer genes [55]. Thus, the only reasonable conclusion here is that FBH1 mutations contribute to the destabilization of the DNA DSB repair machinery in cancer cells.

We next investigated some of the co-occurring mutations in these genes (Table 1, Supplementary Table S3). We find that BRCA2 mutations appear more often than any other gene (Table 1, Supplementary Table S3, Figure 4B). However, as mentioned above, BRCA2 is a very large gene, and this analysis considers any mutation within BRCA2. Additionally, mutations in multiple genes occur most frequently in endometrial and large intestine cancers. We also identified certain FBH1-truncating mutations that are likely to completely inactivate its function. The E900* mutation was detected in a 47-year-old female with cervix squamous cell carcinoma. The same patient also had a homozygous-truncating mutation in BRCA2 (E826*) and upregulation of PALB2 gene expression (Z = 5.638). No mutations or changes in gene expression were detected in BRCA1, RAD52, or RAD51 in this patient. The BRCA2-truncating mutation is in the N-terminus and deletes the RAD51-binding domains, the DNA-binding domains, and the nuclear localization sequences, in essence leading to complete inactivation of the protein product. We identified three other samples with FBH1 truncating mutations that also had an accompanying mutated allele (E668*/F79L; G781*/G756C; and W364*/L582V). The E668*/F79L occurred in a metastatic breast cancer patient (no age information); the G781*/G756C was found in a 53-year-old female with skin malignant melanoma; and the W364*/L582V and G781*/G756C in a female with ductal breast carcinoma (no age information). The E668*/F79L mutation co-occurred with a PALB2 E369Q mutation with no changes in gene expression in BRCA1, BRCA2, RAD52, RAD51, or PALB2. The G781*/G756C co-occurred with a P46H mutation in BRCA1 and a T521= in PALB2, upregulation of BRCA1 gene expression (Z = 2.486) and downregulation of PALB2 gene expression (Z = −2.151).

### 3.4. Structural Analysis of FBH1 Mutations

**Figure 5:**
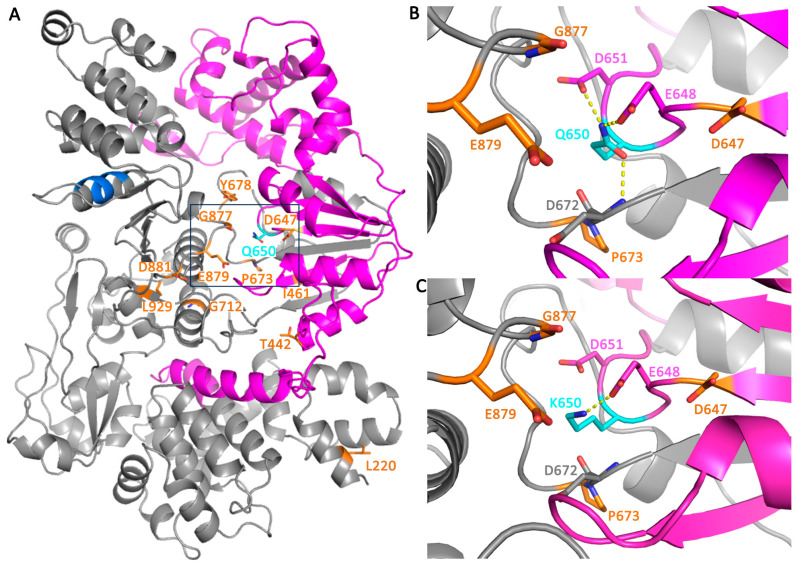
Effect of mutations on the structure of FBH1. (**A**) Overview of FBH1 3D structure model generated from AlphaFold (isoform 2) in which residues 1–215 were removed due to confidence score of less than 70. The UvrD-like helicase ATP-binding domain is shown in magenta, and the APIM domain is shown in blue with the rest of the sequence shown in gray. Gln650 is shown as cyan sticks in the box. All other mutations identified by CHASM to have a *p*-value ≤ 0.1 are shown in orange sticks and labeled. (**B**) Tertiary structure interactions of Q650 and (**C**) of the K650 mutation are shown. Polar interactions (hydrogen bonds and salt bridges) are shown with yellow dashed lines. Side chains of neighboring residues are shown as sticks and labeled. Residues shown in orange have a *p*-value ≤ 0.1.

To understand the 3D location of these mutation on the structure of the protein, an AlphaFold [56] model of FBH1 was used because there is currently no available crystal structure. All mutations identified from CHASM to have a *p* ≤ 0.1 were mapped onto the 3D structure of FBH1 (Figure 5A). The Q650K mutation was analyzed in more detail because it was the only mutation with a *p*-value ≤ 0.05. Six of the eleven mutated residues with a *p* ≤ 0.1 were located within 20 Å of Q650 (I641, D647, P673, Y678, G677, and E879), suggesting this site on the protein is particularly important. An alignment with a structure of UvrD from *Deinococcus radiodurans* (PDB ID: 4C2T) showed that this site of the protein contains the ATP-binding pocket [57]. The Q650K mutation was made computationally using PyMOL and found to disrupt two hydrogen-bonding interactions that stabilize the local tertiary structure of the protein (Figure 5B,C). The mutation of Gln to Lys at position 650 caused some steric clashes, which could also locally destabilize the protein structure. CUPSAT was used to calculate the ΔΔG (kcal/mol) of this mutation, which is a measure of the difference in the free energy of unfolding for the wild-type and mutant protein. A negative value is destabilizing, representing that the mutation reduces the energy barrier for the protein to unfold [58]. Indeed, the CUPSAT analysis of the Q650K mutation predicted it to be destabilizing with a ΔΔG (kcal/mol) of −4.96. Of the possible mutations at this position, a mutation to a lysine residue was predicted to be the most destabilizing.

## 4. Conclusions

Here we queried all FBH1 genetic alterations in human cancers using the data de-posited on COSMIC. Our data show that FBH1 mutations occur in nearly every cancer, while certain cancers are affected by changes in gene expression or copy-number variation, either amplification or reduction in copy number. Although FBH1 has not been classified as a driver gene, the analysis performed here shows that mutations in this gene contribute to the destabilization of the DNA damage repair machinery.

For many of the mutations reported on COSMIC, information on zygosity is not available, and we could not verify (except in a few cases discussed in the report) whether the mutation is homozygous or heterozygous. Thus, a loss of function in one allele of FBH1 may be complemented by a WT allele assuming that the mutation is recessive. However, recent analyses of large databases have uncovered the unexpected finding that even heterozygous mutations can dysregulate the function of a gene. For example, heterozygous mutations in the ATR “tumor suppressor” have been identified that may act in a dominant negative manner analogous to those found in oncogenes [59]. Similar findings were reported for TP53 [60] as well as other genes. Perhaps, most notable are haploinsufficient mutations in the *bona fide* tumor suppressor p27KIP1 [61]. Multiple other examples have been identified as well [62]. Alfred Knudson, the proponent of the two-hit hypothesis, has also refined his assessment of genetic hits in a latter paper [63]. Thus, although we do not have the information for FBH1, it is possible that heterozygous mutations contribute to cancer development for two reasons: they may act as dominant, or they cause haploinsufficiency. Given that much more research has been performed on the consequences of mutations in driver genes, it remains to be determined how mutations in other genes not classified as “driver” promote cellular transformation and immortalization.

## Figures and Tables

**Table 1:** Co-occurring mutations among FBH1, RAD51, RAD52, BRCA1, BRCA2, and PALB2. This is a condensed version of Supplementary Table S3 and includes only those samples that have mutations in both BRCA1 and BRCA2 in addition to FBH1.

Tissue ^a^	FBH1	RAD51	RAD52	BRCA1	BRCA2	PALB2
CNS	C65Y ^b^			K1551R	H264Y, L1357=, N2189=, C393=, E2635=, P2796T, Y3203H	V213=, G112=
CNS	I460T			A1438V, E1033*, N383S	K607=, S973L, E1276*, S1753=, V2010=, G2379R, G2596E, K2833N, T3401M	
CNS	N330S R380W			K1551R	H264Y, L1357=, N2189=, C393=, E2635=, P2796T, Y3203H	V213=, G112=
Endometrium	A546T	A195V		A622V, E733*, L156=	S2835P, Q569H, G25=, P143S, S571F, A1725S, G1761V, A1981S, K2206N, S2216F	R160I
Endometrium	E550K	Z = 2.13 ^c^		R1443Q	S1331Y, H1350N, E1441*	
Endometrium	G928C	A195V		A622V, E733*, L156=	S2835P, Q569H, G25=, P143S, S571F, A1725S, G1761V, A1981S, K2206N, S2216F	R160I
Endometrium	M52L			E1258D, K996Q, E733A	K2316Q	
Endometrium	R975Q	Z = 2.13		R1443Q	S1331Y, H1350N, E1441*	
Endometrium	V274M	A195V		A622V, E733*, L156=	S571F, G25=, P143S, Q569H, A1725S, G1761V, A1981S, K2206N, S2216F, S2835P	R160I
Endometrium	V593I			E597K	S3144Y	L1092=
Endometrium	Y729C	M244I		N665S	N1100S, D191N, C738Y, D980N, K1888E	
Large Intestine	A400V		Z = 2.119	D821Y	S3319Y, F1192C, D479Y, Y2997*	
Large Intestine	G30S			S763F, K1732R, E577*	E2258K, S2052*, L29I, L414=, E1415=	T993=, E554K, R147=
Large Intestine	H202R			N976S, G401E	A1439=, T1505I	T733A, F440Lfs*12
Large Intestine	I512S I989S			K1254T	S3332Y, F701C, L951I, K956T	
Large Intestine	K984T		Z = 2.119	D821Y	S3319Y, F1192C, D479Y, Y2997*	
Large Intestine	S654N			I1318T, K690R, G57=	E97*, D281Y, S445Y, L613R, R645I, L901I, D1352Y, E2635G	P65=
Large Intestine	V1093I		S226F	E349V	E3096K, D3410=	R566H
Skin	D64N			G964V	E215G	
Skin	S1067F			Q1800*	A1253=, G1696E	
Stomach	Q473R			K1497R, T1246A, K1104E	T3033Lfs*29, P190H, L413V	
Upper Aerodigestive	E334K T61N			K1183R, P871L	N372H	
Urinary Tract	E668Q			Q1633H		

^a^ Indicates the cancer type where mutation was observed. This is a condensed summary of Supplemental Table S3. ^b^ Mutation protein coordinates. ^c^ Changes in gene expression where available. Changes are shown in Z-values and only if above Z = 2 or below Z = −2. The “*” symbol represents truncations and the “=” sign silent mutations.

## Data Availability

All data used in this study are available for download on the COSMIC website as described in Section 2.

## Supplementary Materials

The following supporting information can be downloaded at: https://www.mdpi.com/article/10.3390/cancers15184439/s1, Supplementary Table S1: FBH1 mutations reported on COSMIC in all cancer types; Supplementary Table S2: FBH1 mutation analyses; Supplementary Table S3: Co-occurring mutations and gene expression of key recombination genes and FBH1.
