# Supplementary material for: F-box DNA Helicase 1 (FBH1) Contributes to the Destabilization of DNA Damage Repair Machinery in Human Cancers
Source: Cancers (Basel). 2023 Sep 6;15(18):4439. doi: 10.3390/cancers15184439 (PMC10526855; doi:10.3390/cancers15184439)
Supplement: Supplementary file 1 [file cancers-15-04439-s001.zip › Supplementary Table S1.pdf]

Supplementary Table S1. FBH1 mutations reported on COSMIC in all cancer types. References for certain previously published mutations are indicated.

Tissue	FBH1 Mutation Coding	FBH1 Mutation Non-Coding	References
Biliary Tract	p.A226E, p.D833=, p.E141=, p.E529D, p.R1057C, p.R732Q, p.R811W, p.V810L	c.2475+83G>A, c.1550-92G>T, c.310+172dup, c.2875+605_2875+606insCTTATT, c.310+172dup, c.2983-2798dup, c.154+2115G>C, c.310+172dup, c.2983-2798dup, c.677C>A, c.2499C>T, c.423G>A, c.1587G>T, c.3169C>T, c.2195G>A, c.2431C>T, c.2428G>T	[1-5]
Breast	p.A41S, p.A546T, p.E668*, p.E668*, p.F79L, p.G1010=, p.G517S, p.H935L, p.I860M, p.I896M, p.I981M, p.L383=, p.L507=, p.L582, p.N804=, p.N85Efs*31, p.244A, p.Q81*, p.R200W, p.R346Q, p.S159Vfs*102, p.S171F, p.T188P, p.V75=, p.W364*	c.2253+647C>T, c.311-1209C>A, c.2876-18C>T, c.2983-637G>A, c.2983-1449G>A, c.155-34T>C, c.2476-396C>T, c.2983-3270A>G, c.2982+2445G>C, c.2029+5G>C, c.155-2568G>A, c.2254-1166G>T, c.154+662T>A, c.1364+441C>T, c.1365-407G>T, c.2982+1306G>T, c.2982+1304_2982+1309del, c.2254-1068del, c.154+30C>T, c.2254-1G>T, c.121G>T, c.1636G>A, c.2002G>T, c.237C>A, c.3030C>T, c.1549G>A, c.2804A>T, c.2580C>G, c.2688C>G, c.2943C>G, c.1149C>A, c.1521C>G, c.1744C>G, c.2412C>T, c.253_256del, c.730C>G, c.241C>T, c.598C>T, c.1037G>A, c.474del, c.512C>T, c.562A>C, c.225G>A, c.1091G>A	[6-9]
Cervix	p.A809=, p.E258G, p.E900*, p.E963K, p.I545=, p.K520=, p.Q108E, p.Q784=, p.Q81*, p.S261=	c.2427C>T, c.773A>G, c.2698G>T, c.2887G>A, c.1635C>T, c.1560G>A, c.322C>G, c.2352G>A, c.241C>T, c.783C>T	[10]
CNS	p.A217T, p.C65Y, p.G793E, p.G825=, p.I460T, p.I766=, p.L386=, p.N330S, p.P170L, p.R1057H, p.R380W, p.S564=, p.V280L, p.V525A	c.2354-8C>T, c.2254-1196C>T, c.906+1120G>A, c.*254del, c.2116+187G>A, c.2253+1260G>A, c.*253_*254del, c.649G>A, c.194G>A, c.2378G>A, c.2475G>A, c.1379T>C, c.2298C>T, c.1158C>T, c.989A>G, c.509C>T, c.3170G>A, c.1138C>T, c.1692C>T, c.838G>T, c.1574T>C	[11-14]
Endometrium	p.A1062V, p.A198T, p.A439=, p.A546T, p.A809=, p.C36Y, p.C378Y, p.C908Y, p.D932Y, p.E360K, p.E550K, p.G158=, p.G161=, p.G664=, p.G831V, p.G928C, p.I989T, p.L309M, p.M126T, p.M52L, p.P169=, p.P301=, p.Q156R, p.Q851*, p.R1072=, p.R200W, p.R223Q, p.R420=, p.R732=, p.R754W, p.R975Q, p.S564=, p.S617L, p.T76=, p.V274M, p.V290M, p.V539=, p.V593I, p.V639=, p.V746I, p.Y347=, p.Y567=, p.Y729C	c.1458-67G>C, c.154+2457T>A, c.*9C>T, c.-21C>T, c.2551+1G>A, c.*61G>A, c.3185C>T, c.592G>A, c.1317C>T, c.1636G>A, c.2427C>T, c.107G>A, c.1133G>A, c.2723G>A, c.2794G>T, c.1078G>A, c.1648G>A, c.474C>T, c.483G.A, c.1992G>T, c.2492G>T, c.2782G>T, c.2966T>C, c.925C>A, c.377T>C, c.154A>C, c.507G>A, c.903G>A, c.467A>G, c.2551C>T, c.3216C>T, c.598C>T, c.668G>A, c.1258C>A, c.2196G>T, c.2260C>T, c.2924G>A, c.1692C>T, c.1850C>T, c.228C>T, c.820G>A, c.868G>A, c.1617G>T, c.1777G>A, c.1917C>A, c.2236G>A, c.1041C>T, c.1701C>T, c.2186A>G	[15]
Hematopoietic and lymphoid	p.A1073T, p.A168=, p.A546T, p.C1053=, p.D881Y, p.E433A, p.E433K, p.H948Y, p.K775R, p.L398P, p.L507, p.L920=, p.P94=, p.R754W, p.T221=, p.T974S, p.V1016M	c.1037+7T>C, c.2982+1770A>T, c.1365-1237A>G, c.-40G>C, c.22+91C>A, c.1941+156G>A, c.2983-2081T>G, c.155-1436dup, c.1037+7T>C, c.2253+58A>G, c.2117-78A>G, c.1941+99G>A, c.2982+1269A>C, c.2876-689T>G, c.2253+193G>A, c.2982+4064A>G, c.1550-412A>G, c.3217G>A, c.504C>T, c.1636G>A, c.3159T>C, c.2641G>T, c.1298A>C, c.1297G>A, c.2842C>T, c.2324A>G, c.1193T>C, c.1521C>T, c.2758C>T, c.282T>C, c.2260C>T, c.663G>A, c.2921C>G, c.3046G>A	[16-24]

Kidney	p.C353Y, p.G27W, p.I145V, p.I243M, p.S159=, p.V773I,	c.2983-1171G>A, c.2982+551A>G, c.907-938T>C, c.2983-2081T>G, c.2982+784T>A, c.1058G>A, c.79G>T, c.433A>G, c.729T>G, c.477T>C, c.2317G>A	[25,26]
Large intestine	p.A1062V, p.A1073T, p.A185D, p.A377=, p.A400V, p.A41T, p.A50Lfs*3, p.A546T, p.A546T, p.A737T, p.A809=, p.A878T, p.A879=, p.A946V, p.C25=, p.C340R, p.C378Y, p.C770W, p.D125G, p.D385N, p.D658G, p.D790=, p.D914N, p.E550K, p.F536L, p.F79=, p.G101Efs*2, p.G161=, p.G236R, p.G30S, p.G326D, p.G636R, p.G677S, p.G756A, p.G825W, p.H202R, p.H743Pfs*17, p.H909=, p.I1058=, p.I145=, p.I512S, p.I989S, p.K520N, p.K56R, p.K776Rfs*37, p.K984T, p.L1049R, p.L203=, p.L203F, p.L203S, p.L492V, p.L611=, p.N130K, p.N736=, p.N804=, p.P1068Rfs*46, p.P147L, p.P240=, p.P384L, p.P88=, p.P96=, p.Q851*, p.R1013C, p.R1072H, p.R174W, p.R200W, p.R223W, p.R655=, p.R655H, p.R732W, p.R754Q, p.R811Q, p.R820=, p.R848W, p.S178=, p.S40=, p.S654N, p.T1037A, p.T704Nfs*5, p.T750M, p.V1006I, p.V1016=, p.V1025A, p.V1082I, p.V1093I, p.V270D, p.V274M, p.V426M, p.V593I, p.Y436H, p.Y443=, p.Y574F	c.1458-108del, c.2029+20C>T, c.2750-279C>T, c.-27C>T, c.907-107C>T, c.1364+56C>A, c.*49C>T, c.155-126G>A, c.154+162del [25957691], c.2551+145del, c.1365-8del, c.2475+1dup, c.*13C>T, c.1037+1G>A, c.2983-2080G>T, c.2983-2081T>G, c.906+44G>A, c.2029+20C>T, c.*253A>T, c.2750-154C>T, c.2875+627C>A, c.2983-250A>G, c.2876-19del, c.3114+46del, c.1365-8dup, c.1941+2T>C, c.1365-8del, c.2116+2T>C, c.2552-8C>A, c.2749+350G>A, c.2030-18C>T, c.3185C>T, c.3217G>A, c.554C>A, c.1131G>A, c.1199C>T, c.121G>A, c.148del, c.1636G>A, c.2209G>A, c.2427C>T, c.2632G>A, c.2637C>T, c.2837C>T, c.75C>T, c.1018T>C, c.1133G>A, c.2310C>G, c.374A>G, c.1153G>A, c.1973A>G, c.2370C>T, c.2740G>A, c.1648G>A, c.1606T>C, c.237C>T, c.300_301del, c.483G>A, c.706G>A, c.88G>A, c.977G>A, c.1906G>A, c.2029G>A, c.2267G>C, c.2473G>T, c.605A>G, c.2228del, c.2727T>C, c.3174C>T, c.435C>T, c.1535T>G, c.2966T>G, c.1560G>T, c.167A>G, c.2327del, c.2951A>C, c.3146T>G, c.607T>C, c.609G>T, c.608T>C, c.1474C>G, c.1831C>T, c.390T>G, c.2208C>T, c.2412C>T, c.3203del, c.440C>T, c.720T>C, c.1151C>T, c.264G>A, c.288G>A, c.2551C>T, c.3037C>T, c.3215G>A, c.520C>T, c.598C>T, c.667C>T, c.1965C>T, c.1964G>A, c.2194C>T, c.2261G>A, c.2432G>A, c.2460G>A, c.2542C>T, c.534T>G, c.120T>C, c.1961G>A, c.3109A>G, c.2111del, c.2249C>T, c.3016G>A, c.3048G>A, c.3074T>C, c.3244G>A, c.3277G>A, c.809T>A, c.820G>A, c.1276G>A, c.1777G>A, c.1306T>C, c.1329C>T, c.1721A>T	[27-39]
Liver	p.A879=, p.A916=, p.C418=, p.E1079V, p.I624V, p.I707V, p.I835F, p.K173N, p.K526N, p.L68F, p.M126I, p.N501k, p.P96=, p.R849=, p.R849=, p.R97*, p.S1004=, p.S322=, p.T432=, p.T493K, p.V1006I, p.V1012=, p.V399=, p.V627L, p.V711I	c.2476-328A>G, c.2253+786T>G, c.1941+197G>A, c.311-683A>G, c.1941+156G>A, c.155-1072G>C, c.907-835C>T, c.310+42G>T, c.1457+25G>T, c.1549+31G>T, c.3114+5G>T, c.2554-7T>G, c.2117-110G>T, c.22+16G>T, c.2254-24C>A, c.311-39G>T, c1941+133G>A, c.1941+99G>A, c.155-2265A>G, c.2983-977G>T, c.155-1627G>A, c.155-1604C>T, c.1549+198A>T, c.155-2709A>G, c.2254-164A>G, c.2983-2448A>C, c.2983-4115A>C, c.2982+4060A>G, c.310+220A>G, c.2475+31G>A, c.2982+1315A>G, c.2982+1379A>C, c.154+1096T>C, c.154+2656A>G, c.2982+2972T>G, c.2983-2687A>C, c.1942-372T>G, c.155-3050A>G, c.1549+557dup, c.1364+111A>G, c.1719-198G>T, c.1719-199G>T, c.2750-421T>G, c.1174-668A>G, c.155-2612G>A, c.2254-1374A>T, c.2876-160A>C, c.2254-812A>G, c.2254-1351A>G, c.2875+936A>G, c.2983-3747T>C, c.311-795C>T, c.2982+2504T>C, c.-24G>T, c.2637C>T, c.2748A>T, c.1254C>T, c.3236A>T, c.1870A>G, c.2119A>G, c.2503A>T, c.519G>T, c.1578G>T, c.204G>C, c.378G>T, c.1503T>A, c.288G>C, c.2547G>A, c.2547G>A, c.289A>T,	N/A

		c.3012C>T, c.966C>T, c.1296C>T, c.1478C>A, c.3016G>A, c.3036G>T, c.1197G>T, c.1879G>T, c.2131G>A	
Lung	p.A1062E, p.A1088V, p.A50T, p.A50V, p.A546S, p.A791S, p.C1053=, p.C716S, p.D619V, p.D790=, p.D914V, p.E1001*, p.E374D, p.E468D, p.E669K, p.E847Q, p.E885Q, p.E900Q, p.F541=, p.G677C, p.G815W, p.K792E, p.L293F, p.L366= , p.L372=, p.L797M, p.L990=, p.M1031V, p.M126I, p.M144I, p.N343K, p.P206=, p.P279=, p.P301Q, p.P359L, p.P481L, p.P724Q, p.Q120L, p.Q184H, p.Q233R, p.Q37*, p.Q843*, p.R1057S, p.R200Q, p.R211S, p.R455=, p.R535W, p.R551L, p.R979C, p.S150=, p.S482F, p.S713C, p.S752N, p.T877=, p.V1006I, p.V1028=, p.V1093I, p.V509=, p.V805L, p.W191L	c.22+27A>C, c.2551+61A>G, c.906+62C>T, c.2750-153G>A, c.1941+156G>A, c.3114+79G>A, c.1941+99G>A, c.2982+1G>C, c.1458-1G>T, c.2551+1G>T, c.3185C>A, c.3263C>T, c.148G>A, c.149C>T, c.1636G>T, c.2371G>T, c.3159T>C, c.2146T>A, c.1856A>T, c.2370C>T, c.2741A>T, c.3001G>T, c.1122G>T, c.1404G>T, c.2005G>A, c.2539G>C, c.2653G>C, c.2698G>C, c.1623C>T, c.2029G>T, c.2443G>T, c.2374A>G, c.879G>T, C.1098G>C, c.2389T>A, c.2970G>A, c.3091A>G, c.378G>T, c.432G>T, c.1029C>A, c.618A>T, c.837G>A, c.902C>A, c.1076C>T, c.1442C>T, c.2171C>A, c.359A>T, c.552G>T, c.698A>G, c.109C>T, c.2527C>T, c.3169C>A, c.599G>A, c.633G>T, c.1363A>C, c.1603A>T, c.1652G>T, c.2935C>T, c.450C>T, c.1445C>T, c.2138C>G, c.2255G>A, c.2631C>T, c.3016G>A, c.3084C>G, c.3277G>A, c.1527G>T, c.2413G>T, c.572G>T	[40-50]
Nervous System	p.P279L, p.R811W, p.S685=, p.S685I	c.886G>A, c.1798G>A, c.3069C>T, c.836C>T, c.2431C>T, c.2055C>T, c.2054G>T	[9,51,52]
Oesophagus	p.E814K, p.H49=, p.I63T, p.L251=, p.N556=, p.N802=, p.P78=, p.Q14*, p.S534Qfs*62, p.V525=, p.Y875H	c.*254del, c.22+29C>T, c.2982+1955C>A, c.2983-3314C>T, c.2876-690T>G, c.310+1109C>T, c.310+256A>C, c.2983-2526G>C, c.906+62C>T, c.2750-153G>A, c.2440G>A, c.147C>T, c.188T>C, c.753T>C, c.1668C>T, c.2406C>T, c.234C>G, c.40C>T, c.1600_1601del, c.1575C>G, c.2623T>C	[53-57]
Ovary	p.E218G, p.E995Q, p.I629F, p.K792R, p.L980R, p.P240A, p.Q215*, p.Q851H, p.S691=, p.V1011=	c.2876-872C>A, c.2876-689T>G, c.155-1488C>T, c.155-2109A>T, c.2983-2720G>T, c.2253+820C>T, c.154+2594C>G, c.155-2980G>A, c.2983-3005T>A, c.906+936A>C, c.653A>G, c.2983G>C, c.1885A>T, c.2375A>G, c.2939T>G, c.718C>G, c.643C>T, c.2553A>C, c.2073T>C, c.3033G>T	[58,59]
Pancreas	p.E930G, p.R661Q	c.154+562C>T, c.154+1065T>A, c.155-1836C>A, c.2983-2728G>A, c.1942-203C>T, c.2876-386T>A, c.1550-42G>T, c.154+611A>T, c.2253+346G>T, c.311-795C>T, c.155-2148G>A, c.2875+606A>C, c.2983-4285A>G, c.2982+1740A>G, c.2789A<G, c.1982G>A	[1,60]
Prostate	p.A349S, p.C463=, p.D125G, p.H743=, p.812T>C, p.N1005S, p.P1035L, p.P279=, p.P429S, p.R754W, p.R904S, p.T1027=, p.V1093I, p.V1093I, p.V876L	c.1458-7A>T, c.154+2019T>C, c.311-1192T>A, c.154+155T>G, c.3114+126_3114+213del, c.1364+739G>C, c.2875+176G>T, c.3115-30C>A, c.2253+788A>G, c.2983-2080G>T, c.2983-2081T>G, c.154+688_154+695del, c.907-596A>G, c.154+3668G>T, c.2983+395G>A, c.2982+2743C>G, c.2749+140A>G, c.1365-8del, c.1457+107_1457+109del, c.1045G>T, c.1389C>T, c.374A>G, c.2229C>T, c.812T>C, c.3014A>G, c.3104C>T, c.837G>A, c.1285C>T, c.2260C>T, c.2712G>T, c.3081C>A, c.3277G>A, c.3277G>A, c.2626G>T	[61-68]
Skin	p.A400=, p.A549=, p.A889T, p.D232=, p.D64N, p.D693=, p.D698N, p.D932Y, p.E296*, p.E433K, p.E433K, p.E4K,	c.22+2C>A, c.155-1628A>G, c.2983-2078T>G, c.2983-1841T>G, c.2982+2378G>C, c.2982+1948G>A, c.2983-122T>G, c.2749+335G>A, c.2983-1G>T, c.3115-3C>A, c.1200G>T, c.1647C>T,	[69-83]

	p.F1063=, p.F115=, p.F277=, pF303=, p.F588=, p.F817=, p.G1017*, p.G106*, p.G187W, p.G193R, p.G236W p.G30=, p.G391E, p.G391R, p.G677S, p.G756C, p.G781*, p.G781R, p.G80S, p.G91C, p.G921A, p.G928V, p.I1081=, p.I111=, p.I719L, p.I888=, p.L287=, p.L32=, p.L372=, p.L464=, p.L594F, p.L60F, p.L842F, p.L920=, p.N89=, p.P1024S, p.P1068S, p.P147S, p.P255S, p.P279S, p.P305F, p.P305L, p.P305S, p.P355L, p.P373H, p.P724S, p.P742H, p.P78S, p.P844S, p.Q184*, p.Q533H, p.Q701K, p.Q843*, p.R312*, p.R414Q, p.R420=, p.R54L, pR70L, p.R979H, p.S1067F, p.S164L, p.S189=, p.S246L, p.S564=, p.S829L, p.T1037=, p.T188=, p.T39=, p.T521=, p.T730=, p.T730F, p.V403L, p.V440=, p.V943M, p.W11*, p.W294*, p.Y729N	c.2665G>A, c.696C>T, c.190G>A, c.2079C>T, c.2092G>A, c.2794G>T, c.886G>T, c.1297G>A, c.10G>A, c.3189C>T, c.345C>T, c.831C>T, c.909C>T, c.1764C>T, c.2451C>T, c.3049G>T, c.316G>T, c.559G>T, c.577G>A, c.706G>T, c.90C>T, c.1172G>A, c.1171G>A, c.2029G>A, c.2266G>T, c.2341G>T, c.2341G>A, c.238G>A, c.271G>T, c.2762G>C, c.2783G>T, c.3243C>T, c.333C>T, c.2155A>C, c.2664C>T, c.859C>T, c.96C>T, c.1116C>T, c.1390C>T, c.1780C>T, c.178C>T, c.2524C>T, c.2758C>T, c.267C>T, c.3070C>T, c.3202C>T, c.439C>T, c.763C>T, c.835C>T, c.913_914delinsTT, c.914C>T, c.913C>T, c.1064C>T, c.1118C>A, c.2170C>T, c.2225C>A, c.232C>T, c.2530C>T, c.550C>T, c.1599G>T, c.2101C>A, c.2527C>T, c.934C>T, c.1241G>A, c.1260G>A, c.161G>T, c.209G>T, c.2936G>A, c.3200_3201delinsTT, c.491C>T, c.567C>T, c.737C>T, c.1692C>A, c.2486C>T, c.3111C>T, c.564C>T, c.117G>T, c.1563C>T, c.2190C>T, c.2188_2189delinsTT, c.1207G>T, c.1320G>T, c.2827G>A, c.32G>A, c.881G>A, c.2185T>A	
Small Intestine	p.I450F	c.1348A>T	[1]
Soft Tissue	p.F614=, p.L663, p.V1078=	c.2254-668C>T, c.1842C>T, c.1989G>A, c.3234G>A	[84]
Stomach	p.A516, p.A653V, p.A670=, p.A670V, p.A734=, p.C1053=, p.D385N, p.D912V, p.G1010=, p.G27=, p.G449E, p.G91=, p.H273R, p.K530Q, p.L1002=, p.L287M, p.L500=, p.L986S, p.L992F, p.M52I, p.N330S, p.P279Q, p.P954=, p.Q473R, p.Q952=, p.R1057S, p.R190W, p.R551C, p.R754Q, p.R811=, p.S159N, p.S423N, p.T813M, p.V1006I, p.V45=, p.V762A, p.W531S, p.Y387=	c.2876-19del, c.1942-60G>A, c.1549+11C>A, c.1458-108del, c.3114+46del, c.1173+17C>T, c.3114+46del, c.2551+14T>C, c.2254-111dup, c.1458-108del, c.2876-19del, c.2475+41G>A, c.3114+79G>A, c.906+54T>G, c.2116+49G>A, c.2876-19del, c.1458-108del, c.2749+350G>A, c.2551+110C>T, c.2253+52C>T, c.3115-61G>A, c.2749+32dup, c.2551+111G>A, c.154+78G>T, c.2875+305G>A, c.2982+830G>A, c.*25T>C, c.1548C>T, c.1958C>T, c.2010G>A, c.2009C>T, c.2202G>A, c.3159T>C, c.1153G>A, c.2735A>T, c.3030C>T, c.81G>A, c.1346G>A, c.273T>G, c.818A>G, c.1588A>C, c.3006G>A, c.859C>A, c.1500G>A, c.2957T>C , c.2976G>T, c.156G>A, c.989A>G, c.836C>A, c.2862G>A, c.1418A>G, c.2856G>A, c.3169C>A, c.568C>T, c.1651C>T, c.2261G>A, c.2433G>A, c.476G>A, c.1268G>A, c.2438C>T, c.3016G>A, c.135C>G, c.2285T>C, c.1592G>C, c.1161C>T	[56,85-90]
Thyroid	p.A946V, p.F277=, p.F915I	c.2837C>T, c.831C>T, c.2743T>A	
Upper digestive	p.A277V, p.A734V, p.C908=, p.E334K, p.E620=, p.F614Y, p.F615=, p.G239D, p.G519W, p.G763E, p.K1008E, p.L372F, p.P199S, p.P355L, p.P359L, p.Q156*, p.Q508*, p.R103*, p.R312*, p.S246*, p.T61N, p.T623= p.V238L	c.2476-631C>T, c.155-3707dup, c.2876-340G>A, c.3115-86C>T, c.154+3866C>G, c.2983-2092del, c.1130C>T, c.2201C>T, c.2724C>T, c.1000G>A, c.1860A>G, c.1841T>A, c.1845T>C, c.716G>A, c.1555G>T, c.2288G>A, c.3022A>G, c.1114C>T, c.595C>T, c.1064C>T, c.1076C>T, c.466C>T, c.1522C>T, c.306dup, c.934C>T, c.737C>G, c.182C>A, c.1869C>T, c.712G>A	[91-96]
Urinary Tract	p.D300N, p.E374K, p.E668Q, p.F583=, p.F79L, p.G517S, p.L416=, p.L679=, p.L92=, p.N469Y,	c.155-1014G>A, c.155-740C>G, c.1719-248C>G, c.1365-540C>T, c.-26G>T, c.*13C>T, c.2354-10_2360del, c.898G>A, c.1120G>A, c.2002G>C, c.1749C>T, c.237C>A, c.1549G>A, c.1248C>G,	[97,98]

	p.P506R, p.R904M, p.R904M, p.S1067=, p.T348=	c.2037G>A, c.276C>T, c.1405A>T, c.1517C>G, c.2711G>T, c.2711G>T, c.3201C>T, c.1044A>G	

References

1. Gingras, M.C.; Covington, K.R.; Chang, D.K.; Donehower, L.A.; Gill, A.J.; Ittmann, M.M.; Creighton, C.J.; Johns, A.L.; Shinbrot, E.; Dewal, N.; et al. Ampullary Cancers Harbor ELF3 Tumor Suppressor Gene Mutations and Exhibit Frequent WNT Dysregulation. *Cell Rep* **2016**, *14*, 907-919, doi:10.1016/j.celrep.2015.12.005.
2. Jiao, Y.; Pawlik, T.M.; Anders, R.A.; Selaru, F.M.; Streppel, M.M.; Lucas, D.J.; Niknafs, N.; Guthrie, V.B.; Maitra, A.; Argani, P.; et al. Exome sequencing identifies frequent inactivating mutations in BAP1, ARID1A and PBRM1 in intrahepatic cholangiocarcinomas. *Nat Genet* **2013**, *45*, 1470-1473, doi:10.1038/ng.2813.
3. Liu, F.; Li, Y.; Ying, D.; Qiu, S.; He, Y.; Li, M.; Liu, Y.; Zhang, Y.; Zhu, Q.; Hu, Y.; et al. Whole-exome mutational landscape of neuroendocrine carcinomas of the gallbladder. *Signal Transduct Target Ther* **2021**, *6*, 55, doi:10.1038/s41392-020-00412-3.
4. Wardell, C.P.; Fujita, M.; Yamada, T.; Simbolo, M.; Fassan, M.; Karlic, R.; Polak, P.; Kim, J.; Hatanaka, Y.; Maejima, K.; et al. Genomic characterization of biliary tract cancers identifies driver genes and predisposing mutations. *J Hepatol* **2018**, *68*, 959-969, doi:10.1016/j.jhep.2018.01.009.
5. Dong, L.Q.; Shi, Y.; Ma, L.J.; Yang, L.X.; Wang, X.Y.; Zhang, S.; Wang, Z.C.; Duan, M.; Zhang, Z.; Liu, L.Z.; et al. Spatial and temporal clonal evolution of intrahepatic cholangiocarcinoma. *J Hepatol* **2018**, *69*, 89-98, doi:10.1016/j.jhep.2018.02.029.
6. Fu, Y.; Jovelet, C.; Filleron, T.; Pedrero, M.; Motte, N.; Boursin, Y.; Luo, Y.; Massard, C.; Campone, M.; Levy, C.; et al. Improving the Performance of Somatic Mutation Identification by Recovering Circulating Tumor DNA Mutations. *Cancer Res* **2016**, *76*, 5954-5961, doi:10.1158/0008-5472.CAN-15-3457.
7. Lefebvre, C.; Bachelot, T.; Filleron, T.; Pedrero, M.; Campone, M.; Soria, J.C.; Massard, C.; Levy, C.; Arnedos, M.; Lacroix-Triki, M.; et al. Mutational Profile of Metastatic Breast Cancers: A Retrospective Analysis. *PLoS Med* **2016**, *13*, e1002201, doi:10.1371/journal.pmed.1002201.
8. Galante, P.A.; Parmigiani, R.B.; Zhao, Q.; Caballero, O.L.; de Souza, J.E.; Navarro, F.C.; Gerber, A.L.; Nicolas, M.F.; Salim, A.C.; Silva, A.P.; et al. Distinct patterns of somatic alterations in a lymphoblastoid and a tumor genome derived from the same individual. *Nucleic Acids Res* **2011**, *39*, 6056-6068, doi:10.1093/nar/gkr221.
9. Stephens, P.J.; Tarpey, P.S.; Davies, H.; Van Loo, P.; Greenman, C.; Wedge, D.C.; Nik-Zainal, S.; Martin, S.; Varela, I.; Bignell, G.R.; et al. The landscape of cancer genes and mutational processes in breast cancer. *Nature* **2012**, *486*, 400-404, doi:10.1038/nature11017.
10. Jung, S.H.; Choi, Y.J.; Kim, M.S.; Baek, I.P.; Lee, S.H.; Lee, A.W.; Hur, S.Y.; Kim, T.M.; Lee, S.H.; Chung, Y.J. Progression of naive intraepithelial neoplasia genome to aggressive squamous cell carcinoma genome of uterine cervix. *Oncotarget* **2015**, *6*, 4385-4393, doi:10.18632/oncotarget.2981.
11. Lee, J.K.; Wang, J.; Sa, J.K.; Ladewig, E.; Lee, H.O.; Lee, I.H.; Kang, H.J.; Rosenbloom, D.S.; Camara, P.G.; Liu, Z.; et al. Spatiotemporal genomic architecture informs precision oncology in glioblastoma. *Nat Genet* **2017**, *49*, 594-599, doi:10.1038/ng.3806.
12. Patil, V.; Pal, J.; Somasundaram, K. Elucidating the cancer-specific genetic alteration spectrum of glioblastoma derived cell lines from whole exome and RNA sequencing. *Oncotarget* **2015**, *6*, 43452-43471, doi:10.18632/oncotarget.6171.
13. Wu, G.; Diaz, A.K.; Paugh, B.S.; Rankin, S.L.; Ju, B.; Li, Y.; Zhu, X.; Qu, C.; Chen, X.; Zhang, J.; et al. The genomic landscape of diffuse intrinsic pontine glioma and pediatric non-brainstem high-grade glioma. *Nat Genet* **2014**, *46*, 444-450, doi:10.1038/ng.2938.

14. Robinson, G.; Parker, M.; Kranenburg, T.A.; Lu, C.; Chen, X.; Ding, L.; Phoenix, T.N.; Hedlund, E.; Wei, L.; Zhu, X.; et al. Novel mutations target distinct subgroups of medulloblastoma. *Nature* **2012**, *488*, 43-48, doi:10.1038/nature11213.
15. Li, L.; Yue, P.; Song, Q.; Yen, T.T.; Asaka, S.; Wang, T.L.; Beavis, A.L.; Fader, A.N.; Jiao, Y.; Yuan, G.; et al. Genome-wide mutation analysis in precancerous lesions of endometrial carcinoma. *J Pathol* **2021**, *253*, 119-128, doi:10.1002/path.5566.
16. McNerney, M.E.; Brown, C.D.; Peterson, A.L.; Banerjee, M.; Larson, R.A.; Anastasi, J.; Le Beau, M.M.; White, K.P. The spectrum of somatic mutations in high-risk acute myeloid leukaemia with -7/del(7q). *Br J Haematol* **2014**, *166*, 550-556, doi:10.1111/bjh.12964.
17. Gunawardana, J.; Chan, F.C.; Telenius, A.; Woolcock, B.; Kridel, R.; Tan, K.L.; Ben-Neriah, S.; Mottok, A.; Lim, R.S.; Boyle, M.; et al. Recurrent somatic mutations of PTPN1 in primary mediastinal B cell lymphoma and Hodgkin lymphoma. *Nat Genet* **2014**, *46*, 329-335, doi:10.1038/ng.2900.
18. Tessoulin, B.; Moreau-Aubry, A.; Descamps, G.; Gomez-Bougie, P.; Maiga, S.; Gaignard, A.; Chiron, D.; Menoret, E.; Le Gouill, S.; Moreau, P.; et al. Whole-exon sequencing of human myeloma cell lines shows mutations related to myeloma patients at relapse with major hits in the DNA regulation and repair pathways. *J Hematol Oncol* **2018**, *11*, 137, doi:10.1186/s13045-018-0679-0.
19. Roberts, K.G.; Li, Y.; Payne-Turner, D.; Harvey, R.C.; Yang, Y.L.; Pei, D.; McCastlain, K.; Ding, L.; Lu, C.; Song, G.; et al. Targetable kinase-activating lesions in Ph-like acute lymphoblastic leukemia. *N Engl J Med* **2014**, *371*, 1005-1015, doi:10.1056/NEJMoa1403088.
20. Morin, R.D.; Assouline, S.; Alcaide, M.; Mohajeri, A.; Johnston, R.L.; Chong, L.; Grewal, J.; Yu, S.; Fornika, D.; Bushell, K.; et al. Genetic Landscapes of Relapsed and Refractory Diffuse Large B-Cell Lymphomas. *Clin Cancer Res* **2016**, *22*, 2290-2300, doi:10.1158/1078-0432.CCR-15-2123.
21. Wang, L.; Ni, X.; Covington, K.R.; Yang, B.Y.; Shiu, J.; Zhang, X.; Xi, L.; Meng, Q.; Langridge, T.; Drummond, J.; et al. Genomic profiling of Sezary syndrome identifies alterations of key T cell signaling and differentiation genes. *Nat Genet* **2015**, *47*, 1426-1434, doi:10.1038/ng.3444.
22. McGirt, L.Y.; Jia, P.; Baerenwald, D.A.; Duszynski, R.J.; Dahlman, K.B.; Zic, J.A.; Zwerner, J.P.; Hucks, D.; Dave, U.; Zhao, Z.; et al. Whole-genome sequencing reveals oncogenic mutations in mycosis fungoides. *Blood* **2015**, *126*, 508-519, doi:10.1182/blood-2014-11-611194.
23. Nangalia, J.; Massie, C.E.; Baxter, E.J.; Nice, F.L.; Gundem, G.; Wedge, D.C.; Avezov, E.; Li, J.; Kollmann, K.; Kent, D.G.; et al. Somatic CALR mutations in myeloproliferative neoplasms with nonmutated JAK2. *N Engl J Med* **2013**, *369*, 2391-2405, doi:10.1056/NEJMoa1312542.
24. Liu, Y.; Easton, J.; Shao, Y.; Maciaszek, J.; Wang, Z.; Wilkinson, M.R.; McCastlain, K.; Edmonson, M.; Pounds, S.B.; Shi, L.; et al. The genomic landscape of pediatric and young adult T-lineage acute lymphoblastic leukemia. *Nat Genet* **2017**, *49*, 1211-1218, doi:10.1038/ng.3909.
25. Arai, E.; Sakamoto, H.; Ichikawa, H.; Totsuka, H.; Chiku, S.; Gotoh, M.; Mori, T.; Nakatani, T.; Ohnami, S.; Nakagawa, T.; et al. Multilayer-omics analysis of renal cell carcinoma, including the whole exome, methylome and transcriptome. *Int J Cancer* **2014**, *135*, 1330-1342, doi:10.1002/ijc.28768.
26. Sato, Y.; Yoshizato, T.; Shiraishi, Y.; Maekawa, S.; Okuno, Y.; Kamura, T.; Shimamura, T.; Sato-Otsubo, A.; Nagae, G.; Suzuki, H.; et al. Integrated molecular analysis of clear-cell renal cell carcinoma. *Nat Genet* **2013**, *45*, 860-867, doi:10.1038/ng.2699.
27. Mouradov, D.; Sloggett, C.; Jorissen, R.N.; Love, C.G.; Li, S.; Burgess, A.W.; Arango, D.; Strausberg, R.L.; Buchanan, D.; Wormald, S.; et al. Colorectal cancer cell lines are representative models of the main molecular subtypes of primary cancer. *Cancer Res* **2014**, *74*, 3238-3247, doi:10.1158/0008-5472.CAN-14-0013.

28. Giannakis, M.; Mu, X.J.; Shukla, S.A.; Qian, Z.R.; Cohen, O.; Nishihara, R.; Bahl, S.; Cao, Y.; Amin-Mansour, A.; Yamauchi, M.; et al. Genomic Correlates of Immune-Cell Infiltrates in Colorectal Carcinoma. *Cell Rep* **2016**, *15*, 857-865, doi:10.1016/j.celrep.2016.03.075.
29. Cancer Genome Atlas, N. Comprehensive molecular characterization of human colon and rectal cancer. *Nature* **2012**, *487*, 330-337, doi:10.1038/nature11252.
30. Tahara, T.; Yamamoto, E.; Madireddi, P.; Suzuki, H.; Maruyama, R.; Chung, W.; Garriga, J.; Jelinek, J.; Yamano, H.O.; Sugai, T.; et al. Colorectal carcinomas with CpG island methylator phenotype 1 frequently contain mutations in chromatin regulators. *Gastroenterology* **2014**, *146*, 530-538 e535, doi:10.1053/j.gastro.2013.10.060.
31. Giannakis, M.; Hodis, E.; Jasmine Mu, X.; Yamauchi, M.; Rosenbluh, J.; Cibulskis, K.; Saksena, G.; Lawrence, M.S.; Qian, Z.R.; Nishihara, R.; et al. RNF43 is frequently mutated in colorectal and endometrial cancers. *Nat Genet* **2014**, *46*, 1264-1266, doi:10.1038/ng.3127.
32. Yang, J.; Lin, Y.; Huang, Y.; Jin, J.; Zou, S.; Zhang, X.; Li, H.; Feng, T.; Chen, J.; Zuo, Z.; et al. Genome landscapes of rectal cancer before and after preoperative chemoradiotherapy. *Theranostics* **2019**, *9*, 6856-6866, doi:10.7150/thno.37794.
33. Bala, P.; Singh, A.K.; Kavadipula, P.; Kotapalli, V.; Sabarinathan, R.; Bashyam, M.D. Exome sequencing identifies ARID2 as a novel tumor suppressor in early-onset sporadic rectal cancer. *Oncogene* **2021**, *40*, 863-874, doi:10.1038/s41388-020-01537-z.
34. Seshagiri, S.; Stawiski, E.W.; Durinck, S.; Modrusan, Z.; Storm, E.E.; Conboy, C.B.; Chaudhuri, S.; Guan, Y.; Janakiraman, V.; Jaiswal, B.S.; et al. Recurrent R-spondin fusions in colon cancer. *Nature* **2012**, *488*, 660-664, doi:10.1038/nature11282.
35. Sanz-Pamplona, R.; Lopez-Doriga, A.; Pare-Brunet, L.; Lazaro, K.; Bellido, F.; Alonso, M.H.; Ausso, S.; Guino, E.; Beltran, S.; Castro-Giner, F.; et al. Exome Sequencing Reveals AMER1 as a Frequently Mutated Gene in Colorectal Cancer. *Clin Cancer Res* **2015**, *21*, 4709-4718, doi:10.1158/1078-0432.CCR-15-0159.
36. van de Wetering, M.; Francies, H.E.; Francis, J.M.; Bounova, G.; Iorio, F.; Pronk, A.; van Houdt, W.; van Gorp, J.; Taylor-Weiner, A.; Kester, L.; et al. Prospective derivation of a living organoid biobank of colorectal cancer patients. *Cell* **2015**, *161*, 933-945, doi:10.1016/j.cell.2015.03.053.
37. Intarajak, T.; Udomchaiprasertkul, W.; Bunyoo, C.; Yimnoon, J.; Soonklang, K.; Wiriyaukaradecha, K.; Lamlertthon, W.; Sricharunrat, T.; Chaiwiriyawong, W.; Siriphongpreeda, B.; et al. Genetic Aberration Analysis in Thai Colorectal Adenoma and Early-Stage Adenocarcinoma Patients by Whole-Exome Sequencing. *Cancers (Basel)* **2019**, *11*, doi:10.3390/cancers11070977.
38. Kim, T.M.; An, C.H.; Rhee, J.K.; Jung, S.H.; Lee, S.H.; Baek, I.P.; Kim, M.S.; Lee, S.H.; Chung, Y.J. Clonal origins and parallel evolution of regionally synchronous colorectal adenoma and carcinoma. *Oncotarget* **2015**, *6*, 27725-27735, doi:10.18632/oncotarget.4834.
39. Bertotti, A.; Papp, E.; Jones, S.; Adleff, V.; Anagnostou, V.; Lupo, B.; Sausen, M.; Phallen, J.; Hruban, C.A.; Tokheim, C.; et al. The genomic landscape of response to EGFR blockade in colorectal cancer. *Nature* **2015**, *526*, 263-267, doi:10.1038/nature14969.
40. Krause, A.; Roma, L.; Lorber, T.; Habicht, J.; Lardinois, D.; De Filippo, M.R.; Prince, S.S.; Piscuoglio, S.; Ng, C.; Bubendorf, L. Deciphering the clonal relationship between glandular and squamous components in adenosquamous carcinoma of the lung using whole exome sequencing. *Lung Cancer* **2020**, *150*, 132-138, doi:10.1016/j.lungcan.2020.10.013.
41. McMillan, E.A.; Ryu, M.J.; Diep, C.H.; Mendiratta, S.; Clemenceau, J.R.; Vaden, R.M.; Kim, J.H.; Motoyaji, T.; Covington, K.R.; Peyton, M.; et al. Chemistry-First Approach for Nomination of Personalized Treatment in Lung Cancer. *Cell* **2018**, *173*, 864-878 e829, doi:10.1016/j.cell.2018.03.028.

42. Wagner, A.H.; Devarakonda, S.; Skidmore, Z.L.; Krysiak, K.; Ramu, A.; Trani, L.; Kunisaki, J.; Masood, A.; Waqar, S.N.; Spies, N.C.; et al. Recurrent WNT pathway alterations are frequent in relapsed small cell lung cancer. *Nat Commun* **2018**, *9*, 3787, doi:10.1038/s41467-018-06162-9.
43. George, J.; Walter, V.; Peifer, M.; Alexandrov, L.B.; Seidel, D.; Leenders, F.; Maas, L.; Muller, C.; Dahmen, I.; Delhomme, T.M.; et al. Integrative genomic profiling of large-cell neuroendocrine carcinomas reveals distinct subtypes of high-grade neuroendocrine lung tumors. *Nat Commun* **2018**, *9*, 1048, doi:10.1038/s41467-018-03099-x.
44. Imielinski, M.; Berger, A.H.; Hammerman, P.S.; Hernandez, B.; Pugh, T.J.; Hodis, E.; Cho, J.; Suh, J.; Capelletti, M.; Sivachenko, A.; et al. Mapping the hallmarks of lung adenocarcinoma with massively parallel sequencing. *Cell* **2012**, *150*, 1107-1120, doi:10.1016/j.cell.2012.08.029.
45. Shi, J.; Hua, X.; Zhu, B.; Ravichandran, S.; Wang, M.; Nguyen, C.; Brodie, S.A.; Palleschi, A.; Alloisio, M.; Pariscenti, G.; et al. Somatic Genomics and Clinical Features of Lung Adenocarcinoma: A Retrospective Study. *PLoS Med* **2016**, *13*, e1002162, doi:10.1371/journal.pmed.1002162.
46. Li, C.; Gao, Z.; Li, F.; Li, X.; Sun, Y.; Wang, M.; Li, D.; Wang, R.; Li, F.; Fang, R.; et al. Whole Exome Sequencing Identifies Frequent Somatic Mutations in Cell-Cell Adhesion Genes in Chinese Patients with Lung Squamous Cell Carcinoma. *Sci Rep* **2015**, *5*, 14237, doi:10.1038/srep14237.
47. Boora, G.K.; Kanwar, R.; Kulkarni, A.A.; Pleticha, J.; Ames, M.; Schroth, G.; Beutler, A.S.; Banck, M.S. Exome-level comparison of primary well-differentiated neuroendocrine tumors and their cell lines. *Cancer Genet* **2015**, *208*, 374-381, doi:10.1016/j.cancergen.2015.04.002.
48. George, J.; Lim, J.S.; Jang, S.J.; Cun, Y.; Ozretic, L.; Kong, G.; Leenders, F.; Lu, X.; Fernandez-Cuesta, L.; Bosco, G.; et al. Comprehensive genomic profiles of small cell lung cancer. *Nature* **2015**, *524*, 47-53, doi:10.1038/nature14664.
49. Liu, J.; Lee, W.; Jiang, Z.; Chen, Z.; Jhunjhunwala, S.; Haverty, P.M.; Gnad, F.; Guan, Y.; Gilbert, H.N.; Stinson, J.; et al. Genome and transcriptome sequencing of lung cancers reveal diverse mutational and splicing events. *Genome Res* **2012**, *22*, 2315-2327, doi:10.1101/gr.140988.112.
50. Rudin, C.M.; Durinck, S.; Stawiski, E.W.; Poirier, J.T.; Modrusan, Z.; Shames, D.S.; Bergbower, E.A.; Guan, Y.; Shin, J.; Guillory, J.; et al. Comprehensive genomic analysis identifies SOX2 as a frequently amplified gene in small-cell lung cancer. *Nat Genet* **2012**, *44*, 1111-1116, doi:10.1038/ng.2405.
51. Van Allen, E.M.; Wagle, N.; Sucker, A.; Treacy, D.J.; Johannessen, C.M.; Goetz, E.M.; Place, C.S.; Taylor-Weiner, A.; Whittaker, S.; Kryukov, G.V.; et al. The genetic landscape of clinical resistance to RAF inhibition in metastatic melanoma. *Cancer Discov* **2014**, *4*, 94-109, doi:10.1158/2159-8290.CD-13-0617.
52. Dutton-Regester, K.; Kakavand, H.; Aoude, L.G.; Stark, M.S.; Gartside, M.G.; Johansson, P.; O'Connor, L.; Lanagan, C.; Tembe, V.; Pupo, G.M.; et al. Melanomas of unknown primary have a mutation profile consistent with cutaneous sun-exposed melanoma. *Pigment Cell Melanoma Res* **2013**, *26*, 852-860, doi:10.1111/pcmr.12153.
53. Zhang, L.; Zhou, Y.; Cheng, C.; Cui, H.; Cheng, L.; Kong, P.; Wang, J.; Li, Y.; Chen, W.; Song, B.; et al. Genomic analyses reveal mutational signatures and frequently altered genes in esophageal squamous cell carcinoma. *Am J Hum Genet* **2015**, *96*, 597-611, doi:10.1016/j.ajhg.2015.02.017.
54. Cheng, C.; Cui, H.; Zhang, L.; Jia, Z.; Song, B.; Wang, F.; Li, Y.; Liu, J.; Kong, P.; Shi, R.; et al. Genomic analyses reveal FAM84B and the NOTCH pathway are associated with the progression of esophageal squamous cell carcinoma. *Gigascience* **2016**, *5*, 1, doi:10.1186/s13742-015-0107-0.
55. Sawada, G.; Niida, A.; Uchi, R.; Hirata, H.; Shimamura, T.; Suzuki, Y.; Shiraishi, Y.; Chiba, K.; Imoto, S.; Takahashi, Y.; et al. Genomic Landscape of Esophageal Squamous Cell Carcinoma in a Japanese Population. *Gastroenterology* **2016**, *150*, 1171-1182, doi:10.1053/j.gastro.2016.01.035.

56. Dulak, A.M.; Stojanov, P.; Peng, S.; Lawrence, M.S.; Fox, C.; Stewart, C.; Bandla, S.; Imamura, Y.; Schumacher, S.E.; Shefler, E.; et al. Exome and whole-genome sequencing of esophageal adenocarcinoma identifies recurrent driver events and mutational complexity. *Nat Genet* **2013**, *45*, 478-486, doi:10.1038/ng.2591.
57. Gao, Y.B.; Chen, Z.L.; Li, J.G.; Hu, X.D.; Shi, X.J.; Sun, Z.M.; Zhang, F.; Zhao, Z.R.; Li, Z.T.; Liu, Z.Y.; et al. Genetic landscape of esophageal squamous cell carcinoma. *Nat Genet* **2014**, *46*, 1097-1102, doi:10.1038/ng.3076.
58. Cancer Genome Atlas Research, N. Integrated genomic analyses of ovarian carcinoma. *Nature* **2011**, *474*, 609-615, doi:10.1038/nature10166.
59. Cheasley, D.; Wakefield, M.J.; Ryland, G.L.; Allan, P.E.; Alsop, K.; Amarasinghe, K.C.; Ananda, S.; Anglesio, M.S.; Au-Yeung, G.; Bohm, M.; et al. The molecular origin and taxonomy of mucinous ovarian carcinoma. *Nat Commun* **2019**, *10*, 3935, doi:10.1038/s41467-019-11862-x.
60. Jakel, C.; Bergmann, F.; Toth, R.; Assenov, Y.; van der Duin, D.; Strobel, O.; Hank, T.; Kloppel, G.; Dorrell, C.; Grompe, M.; et al. Genome-wide genetic and epigenetic analyses of pancreatic acinar cell carcinomas reveal aberrations in genome stability. *Nat Commun* **2017**, *8*, 1323, doi:10.1038/s41467-017-01118-x.
61. Kumar, A.; Coleman, I.; Morrissey, C.; Zhang, X.; True, L.D.; Gulati, R.; Etzioni, R.; Bolouri, H.; Montgomery, B.; White, T.; et al. Substantial interindividual and limited intraindividual genomic diversity among tumors from men with metastatic prostate cancer. *Nat Med* **2016**, *22*, 369-378, doi:10.1038/nm.4053.
62. Berger, M.F.; Lawrence, M.S.; Demichelis, F.; Drier, Y.; Cibulskis, K.; Sivachenko, A.Y.; Sboner, A.; Esgueva, R.; Pflueger, D.; Sougnez, C.; et al. The genomic complexity of primary human prostate cancer. *Nature* **2011**, *470*, 214-220, doi:10.1038/nature09744.
63. Robinson, D.; Van Allen, E.M.; Wu, Y.M.; Schultz, N.; Lonigro, R.J.; Mosquera, J.M.; Montgomery, B.; Taplin, M.E.; Pritchard, C.C.; Attard, G.; et al. Integrative clinical genomics of advanced prostate cancer. *Cell* **2015**, *161*, 1215-1228, doi:10.1016/j.cell.2015.05.001.
64. Grasso, C.S.; Wu, Y.M.; Robinson, D.R.; Cao, X.; Dhanasekaran, S.M.; Khan, A.P.; Quist, M.J.; Jing, X.; Lonigro, R.J.; Brenner, J.C.; et al. The mutational landscape of lethal castration-resistant prostate cancer. *Nature* **2012**, *487*, 239-243, doi:10.1038/nature11125.
65. Petrovics, G.; Li, H.; Stumpel, T.; Tan, S.H.; Young, D.; Katta, S.; Li, Q.; Ying, K.; Klocke, B.; Ravindranath, L.; et al. A novel genomic alteration of LSAMP associates with aggressive prostate cancer in African American men. *EBioMedicine* **2015**, *2*, 1957-1964, doi:10.1016/j.ebiom.2015.10.028.
66. Chen, E.J.; Sowalsky, A.G.; Gao, S.; Cai, C.; Voznesensky, O.; Schaefer, R.; Loda, M.; True, L.D.; Ye, H.; Troncoso, P.; et al. Abiraterone treatment in castration-resistant prostate cancer selects for progesterone responsive mutant androgen receptors. *Clin Cancer Res* **2015**, *21*, 1273-1280, doi:10.1158/1078-0432.CCR-14-1220.
67. Lindberg, J.; Klevebring, D.; Liu, W.; Neiman, M.; Xu, J.; Wiklund, P.; Wiklund, F.; Mills, I.G.; Egevad, L.; Gronberg, H. Exome sequencing of prostate cancer supports the hypothesis of independent tumour origins. *Eur Urol* **2013**, *63*, 347-353, doi:10.1016/j.eururo.2012.03.050.
68. Lindberg, J.; Mills, I.G.; Klevebring, D.; Liu, W.; Neiman, M.; Xu, J.; Wikstrom, P.; Wiklund, P.; Wiklund, F.; Egevad, L.; et al. The mitochondrial and autosomal mutation landscapes of prostate cancer. *Eur Urol* **2013**, *63*, 702-708, doi:10.1016/j.eururo.2012.11.053.
69. Shain, A.H.; Garrido, M.; Botton, T.; Talevich, E.; Yeh, I.; Sanborn, J.Z.; Chung, J.; Wang, N.J.; Kakavand, H.; Mann, G.J.; et al. Exome sequencing of desmoplastic melanoma identifies recurrent NFKBIE promoter mutations and diverse activating mutations in the MAPK pathway. *Nat Genet* **2015**, *47*, 1194-1199, doi:10.1038/ng.3382.

70. Bonilla, X.; Parmentier, L.; King, B.; Bezrukov, F.; Kaya, G.; Zoete, V.; Seplyarskiy, V.B.; Sharpe, H.J.; McKee, T.; Letourneau, A.; et al. Genomic analysis identifies new drivers and progression pathways in skin basal cell carcinoma. *Nat Genet* **2016**, *48*, 398-406, doi:10.1038/ng.3525.
71. Rabbie, R.; Ferguson, P.; Wong, K.; Couturier, D.L.; Moran, U.; Turner, C.; Emanuel, P.; Haas, K.; Saunus, J.M.; Davidson, M.R.; et al. The mutational landscape of melanoma brain metastases presenting as the first visceral site of recurrence. *Br J Cancer* **2021**, *124*, 156-160, doi:10.1038/s41416-020-01090-2.
72. Hintzsche, J.D.; Gorden, N.T.; Amato, C.M.; Kim, J.; Wuensch, K.E.; Robinson, S.E.; Applegate, A.J.; Couts, K.L.; Medina, T.M.; Wells, K.R.; et al. Whole-exome sequencing identifies recurrent SF3B1 R625 mutation and comutation of NF1 and KIT in mucosal melanoma. *Melanoma Res* **2017**, *27*, 189-199, doi:10.1097/CMR.0000000000000345.
73. Hayward, N.K.; Wilmott, J.S.; Waddell, N.; Johansson, P.A.; Field, M.A.; Nones, K.; Patch, A.M.; Kakavand, H.; Alexandrov, L.B.; Burke, H.; et al. Whole-genome landscapes of major melanoma subtypes. *Nature* **2017**, *545*, 175-180, doi:10.1038/nature22071.
74. Wilmott, J.S.; Johansson, P.A.; Newell, F.; Waddell, N.; Ferguson, P.; Quek, C.; Patch, A.M.; Nones, K.; Shang, P.; Pritchard, A.L.; et al. Whole genome sequencing of melanomas in adolescent and young adults reveals distinct mutation landscapes and the potential role of germline variants in disease susceptibility. *Int J Cancer* **2019**, *144*, 1049-1060, doi:10.1002/ijc.31791.
75. Pickering, C.R.; Zhou, J.H.; Lee, J.J.; Drummond, J.A.; Peng, S.A.; Saade, R.E.; Tsai, K.Y.; Curry, J.L.; Tetzlaff, M.T.; Lai, S.Y.; et al. Mutational landscape of aggressive cutaneous squamous cell carcinoma. *Clin Cancer Res* **2014**, *20*, 6582-6592, doi:10.1158/1078-0432.CCR-14-1768.
76. South, A.P.; Purdie, K.J.; Watt, S.A.; Haldenby, S.; den Breems, N.; Dimon, M.; Arron, S.T.; Kluk, M.J.; Aster, J.C.; McHugh, A.; et al. NOTCH1 mutations occur early during cutaneous squamous cell carcinogenesis. *J Invest Dermatol* **2014**, *134*, 2630-2638, doi:10.1038/jid.2014.154.
77. Sanborn, J.Z.; Chung, J.; Purdom, E.; Wang, N.J.; Kakavand, H.; Wilmott, J.S.; Butler, T.; Thompson, J.F.; Mann, G.J.; Haydu, L.E.; et al. Phylogenetic analyses of melanoma reveal complex patterns of metastatic dissemination. *Proc Natl Acad Sci U S A* **2015**, *112*, 10995-11000, doi:10.1073/pnas.1508074112.
78. Sharpe, H.J.; Pau, G.; Dijkgraaf, G.J.; Basset-Seguin, N.; Modrusan, Z.; Januario, T.; Tsui, V.; Durham, A.B.; Dlugosz, A.A.; Haverty, P.M.; et al. Genomic analysis of smoothened inhibitor resistance in basal cell carcinoma. *Cancer Cell* **2015**, *27*, 327-341, doi:10.1016/j.ccell.2015.02.001.
79. Krauthammer, M.; Kong, Y.; Bacchiocchi, A.; Evans, P.; Pornputtapong, N.; Wu, C.; McCusker, J.P.; Ma, S.; Cheng, E.; Straub, R.; et al. Exome sequencing identifies recurrent mutations in NF1 and RASopathy genes in sun-exposed melanomas. *Nat Genet* **2015**, *47*, 996-1002, doi:10.1038/ng.3361.
80. Berger, M.F.; Hodis, E.; Heffernan, T.P.; Deribe, Y.L.; Lawrence, M.S.; Protopopov, A.; Ivanova, E.; Watson, I.R.; Nickerson, E.; Ghosh, P.; et al. Melanoma genome sequencing reveals frequent PREX2 mutations. *Nature* **2012**, *485*, 502-506, doi:10.1038/nature11071.
81. Wong, K.; van der Weyden, L.; Schott, C.R.; Foote, A.; Constantino-Casas, F.; Smith, S.; Dobson, J.M.; Murchison, E.P.; Wu, H.; Yeh, I.; et al. Cross-species genomic landscape comparison of human mucosal melanoma with canine oral and equine melanoma. *Nat Commun* **2019**, *10*, 353, doi:10.1038/s41467-018-08081-1.
82. Krauthammer, M.; Kong, Y.; Ha, B.H.; Evans, P.; Bacchiocchi, A.; McCusker, J.P.; Cheng, E.; Davis, M.J.; Goh, G.; Choi, M.; et al. Exome sequencing identifies recurrent somatic RAC1 mutations in melanoma. *Nat Genet* **2012**, *44*, 1006-1014, doi:10.1038/ng.2359.
83. Durinck, S.; Ho, C.; Wang, N.J.; Liao, W.; Jakkula, L.R.; Collisson, E.A.; Pons, J.; Chan, S.W.; Lam, E.T.; Chu, C.; et al. Temporal dissection of tumorigenesis in primary cancers. *Cancer Discov* **2011**, *1*, 137-143, doi:10.1158/2159-8290.CD-11-0028.

84. Shankar, G.M.; Taylor-Weiner, A.; Lelic, N.; Jones, R.T.; Kim, J.C.; Francis, J.M.; Abedalthagafi, M.; Borges, L.F.; Coumans, J.V.; Curry, W.T.; et al. Sporadic hemangioblastomas are characterized by cryptic VHL inactivation. *Acta Neuropathol Commun* **2014**, *2*, 167, doi:10.1186/s40478-014-0167-x.
85. Lim, C.H.; Cho, Y.K.; Kim, S.W.; Choi, M.G.; Rhee, J.K.; Chung, Y.J.; Lee, S.H.; Kim, T.M. The chronological sequence of somatic mutations in early gastric carcinogenesis inferred from multiregion sequencing of gastric adenomas. *Oncotarget* **2016**, *7*, 39758-39767, doi:10.18632/oncotarget.9250.
86. Wang, K.; Yuen, S.T.; Xu, J.; Lee, S.P.; Yan, H.H.; Shi, S.T.; Siu, H.C.; Deng, S.; Chu, K.M.; Law, S.; et al. Whole-genome sequencing and comprehensive molecular profiling identify new driver mutations in gastric cancer. *Nat Genet* **2014**, *46*, 573-582, doi:10.1038/ng.2983.
87. Seidlitz, T.; Merker, S.R.; Rothe, A.; Zakrzewski, F.; von Neubeck, C.; Grutzmann, K.; Sommer, U.; Schweitzer, C.; Scholch, S.; Uhlemann, H.; et al. Human gastric cancer modelling using organoids. *Gut* **2019**, *68*, 207-217, doi:10.1136/gutjnl-2017-314549.
88. Kim, T.M.; Jung, S.H.; Kim, M.S.; Baek, I.P.; Park, S.W.; Lee, S.H.; Lee, H.H.; Kim, S.S.; Chung, Y.J.; Lee, S.H. The mutational burdens and evolutionary ages of early gastric cancers are comparable to those of advanced gastric cancers. *J Pathol* **2014**, *234*, 365-374, doi:10.1002/path.4401.
89. Liu, J.; McCleland, M.; Stawiski, E.W.; Gnad, F.; Mayba, O.; Haverty, P.M.; Durinck, S.; Chen, Y.J.; Klijn, C.; Jhunjhunwala, S.; et al. Integrated exome and transcriptome sequencing reveals ZAK isoform usage in gastric cancer. *Nat Commun* **2014**, *5*, 3830, doi:10.1038/ncomms4830.
90. Lim, B.; Kim, C.; Kim, J.H.; Kwon, W.S.; Lee, W.S.; Kim, J.M.; Park, J.Y.; Kim, H.S.; Park, K.H.; Kim, T.S.; et al. Genetic alterations and their clinical implications in gastric cancer peritoneal carcinomatosis revealed by whole-exome sequencing of malignant ascites. *Oncotarget* **2016**, *7*, 8055-8066, doi:10.18632/oncotarget.6977.
91. Li, Y.Y.; Chung, G.T.; Lui, V.W.; To, K.F.; Ma, B.B.; Chow, C.; Woo, J.K.; Yip, K.Y.; Seo, J.; Hui, E.P.; et al. Exome and genome sequencing of nasopharynx cancer identifies NF-kappaB pathway activating mutations. *Nat Commun* **2017**, *8*, 14121, doi:10.1038/ncomms14121.
92. Zhang, L.; MacIsaac, K.D.; Zhou, T.; Huang, P.Y.; Xin, C.; Dobson, J.R.; Yu, K.; Chiang, D.Y.; Fan, Y.; Pelletier, M.; et al. Genomic Analysis of Nasopharyngeal Carcinoma Reveals TME-Based Subtypes. *Mol Cancer Res* **2017**, *15*, 1722-1732, doi:10.1158/1541-7786.MCR-17-0134.
93. India Project Team of the International Cancer Genome, C. Mutational landscape of gingivo-buccal oral squamous cell carcinoma reveals new recurrently-mutated genes and molecular subgroups. *Nat Commun* **2013**, *4*, 2873, doi:10.1038/ncomms3873.
94. Al-Hebshi, N.N.; Li, S.; Nasher, A.T.; El-Setouhy, M.; Alsanosi, R.; Blancato, J.; Loffredo, C. Exome sequencing of oral squamous cell carcinoma in users of Arabian snuff reveals novel candidates for driver genes. *Int J Cancer* **2016**, *139*, 363-372, doi:10.1002/ijc.30068.
95. Pickering, C.R.; Zhang, J.; Yoo, S.Y.; Bengtsson, L.; Moorthy, S.; Neskey, D.M.; Zhao, M.; Ortega Alves, M.V.; Chang, K.; Drummond, J.; et al. Integrative genomic characterization of oral squamous cell carcinoma identifies frequent somatic drivers. *Cancer Discov* **2013**, *3*, 770-781, doi:10.1158/2159-8290.CD-12-0537.
96. Fadlullah, M.Z.; Chiang, I.K.; Dionne, K.R.; Yee, P.S.; Gan, C.P.; Sam, K.K.; Tiong, K.H.; Ng, A.K.; Martin, D.; Lim, K.P.; et al. Genetically-defined novel oral squamous cell carcinoma cell lines for the development of molecular therapies. *Oncotarget* **2016**, *7*, 27802-27818, doi:10.18632/oncotarget.8533.

97. Lawson, A.R.J.; Abascal, F.; Coorens, T.H.H.; Hooks, Y.; O'Neill, L.; Latimer, C.; Raine, K.; Sanders, M.A.; Warren, A.Y.; Mahbubani, K.T.A.; et al. Extensive heterogeneity in somatic mutation and selection in the human bladder. *Science* **2020**, *370*, 75-82, doi:10.1126/science.aba8347.
98. Van Allen, E.M.; Mouw, K.W.; Kim, P.; Iyer, G.; Wagle, N.; Al-Ahmadie, H.; Zhu, C.; Ostrovnaya, I.; Kryukov, G.V.; O'Connor, K.W.; et al. Somatic ERCC2 mutations correlate with cisplatin sensitivity in muscle-invasive urothelial carcinoma. *Cancer Discov* **2014**, *4*, 1140-1153, doi:10.1158/2159-8290.CD-14-0623.
