# Supplementary material for: F-box DNA Helicase 1 (FBH1) Contributes to the Destabilization of DNA Damage Repair Machinery in Human Cancers
Source: Cancers (Basel). 2023 Sep 6;15(18):4439. doi: 10.3390/cancers15184439 (PMC10526855; doi:10.3390/cancers15184439)
Supplement: Supplementary file 1 [file cancers-15-04439-s001.zip › Supplementary Table S3.pdf]

Supplementary Table S3. Co-occurring mutations and gene expression of key recombination genes and FBH1. Changes in gene expression are only available for TCGA samples and are expressed in Z values.

Tissue	FBH1 Mutation	RAD51		RAD52		BRCA1		BRCA2		PALB2	
		MUT	GE	MUT	GE	MUT	GE	MUT	GE	MUT	GE
Biliary Tract	A226E							1784Tfs *7			
Biliary Tract	D833=									D1177 Mfs*	
Biliary Tract	E141=			S251=		S1494Ff s*, Q1135=, I1068S		D224N, K2162=			
Biliary Tract	R732Q							R2949 M			
Biliary Tract	V810L							P3202L			
Breast	A546T						2.494				
Breast	E668*									E639Q	
Breast	F79L									E639Q	
Breast	I860M								2.293		2.515
Breast	I896M								2.293		2.515
Breast	I981M										2.146
Breast	L383=								2.169		
Breast	N85Efs*31				2.494						
Breast	P244A								5.504		3.442
Breast	Q81*										2.508
Breast	R200W							760Pfs* 13			
Breast	R346Q		6.715								
Breast	T188P										2.049
CNS	A217T		7.184				2.386	W2970*	2.494		2.279

CNS	C65Y					K1551R		H264Y, L1357=, N2189=		V213=, G112=	
CNS	C65Y					K1551R		H264Y, L1357=, N2189=, C393=, E2635=, P2796T, Y3203H		V213=, G112=	
CNS	G825=					K1551R		H264Y, L1357=, N2189=		V213=, G112=	
CNS	G825=					K1551R		H264Y, L1357=, N2189=, C393=, E2635=, P2796T, Y3203H		V213=, G112=	
CNS	I460T					A1438V, E1033*, N383S		K607=, S973L, E1276*, S1753=, V2010=, G2379R, G2596E, K2833N, T3401M			

CNS	I766=							R107=, E3223K			
CNS	L386=					S1882N					
CNS	N330S					K1551R		H264Y, L1357=, N2189=		V213=, G112=	
CNS	N330S					K1551R		H264Y, L1357=, N2189= , C393=, E2635=, P2796T, Y3203H		V213=, G112=	
CNS	P170L							R107=, E3223K			
CNS	R1057H				2.521						
CNS	R380W					K1551R		H264Y, L1357=, N2189=		V213=, G112=	
CNS	R380W					K1551R		H264Y, L1357=, N2189= , C393=, E2635=, P2796T, Y3203H		V213=, G112=	
CNS	S564=					K1551R		H254Y, L1357=, N2189=		V213=, G112=	
Cervix	A809=					E1703K					
Cervix	E258G						-2.44				
Cervix	E900*							E826*			5.638
Cervix	Q108E						2.212				

Cervix	Q784=									L961=	2.36
Endometrium	A1062V	A329V								S835=, V410A, A406V	
Endometrium	A198T							T1887M, S196I, T868I, I1772S, L2289=, S3239Y		M1067T, A291V	
Endometrium	A439=		2.613				2.151				
Endometrium	A546T	A195V				A622V, E733*, L156=		S2835P, Q569H, G25=, P143S, S571F, A1725S, G1761V, A1981S, K2206N, S2216F		R160I	
Endometrium	A809=			R260=			2.393	A518V, A1896T			
Endometrium	C36Y			S229N						R37C	
Endometrium	C908Y			A146V			2.587	E1441*, G405R			
Endometrium	D932Y					V740=	3.672	N20D, E386A,		P358L	

								E514G, G1122*, S2773Y			
Endometrium	E360K							G904*			
Endometrium	E550K		2.13			R1443Q		S1331Y, H1350N, E1441*			
Endometrium	G158=							E2020K, N2119=, T2542M		A946T	
Endometrium	G161=	M244I				N665S		N1100S, D191N, C738Y, D980N, K1888E			
Endometrium	G664=							G904*			
Endometrium	G831V							K16E, T867I	2.334		
Endometrium	G928C	A195V				A622V, E733*, L156=		S2835P, Q569H, G25=, P143S, S571F, A1725S, G1761V, A1981S,		R160I	

								K2206N, S2216F			
Endometrium	L309M					V740=	3.672	N20D, E386A, E514G, G1122*, S2773Y		P358L	
Endometrium	M126T							P512H			
Endometrium	M52L					E1258D, K996Q, E733A		K2316Q			
Endometrium	P169=							E2020K, N2119=, T2542M		A946T	
Endometrium	R1072=					R1443*, T1618=, A942V		Q3247H			
Endometrium	R223Q			R260=			2.393	A1896T			
Endometrium	R420=	A329V								S835=, V410A, A406V	
Endometrium	R732=							G904*			
Endometrium	R754W			S373=		A1082=		N1603S, W1692Mfs*3, K1863N,		A968V	

								C2212W, R3007I			
Endometrium	R754W			S373=		A1082=		N1603S, W1692Mfs*3, K1863N, C2212W, R3007I		A968V	
Endometrium	R975Q		2.13			R1443Q		S1331Y, H1350N, E1441*			
Endometrium	E18K							T1483P			
Endometrium	T76=	M2441				N665S		N1100S, D191N, C738Y, D980N, K1888E			
Endometrium	V274M	A195V				A622V, E733*, L156=		S571F, G25=, P143S, Q569H, A1725S, G1761V, A1981S, K2206		R160I	

								N, S2216F, S2835P			
Endometrium	V290M		2.212	G399*, M78I			2.077	K2472=, Q2870=, F3273L			
Endometrium	V539=					E673*		S1172L, S1680Y, S3396R	2.191		2.008
Endometrium	V593I					E597K		S3144Y		L1092=	
Endometrium	V639=							E510*, E897*, L557V, L759I, E2476*			
Endometrium	Y347=					R1443*, T1618=, A942V		R2842C, F1192C, N1747S, T2125Nfs*4, D2438=, A2711T, Q3247H			
Endometrium	Y567=					S1180I, L574I, L49M		T1067A, V1078I, T2880I		A736=	
Endometrium	Y729C	M244I				N665S		N1100S, D191N,			

								C738Y, D980N, K1888E			
Hemopoietic and Lymphoid Tissue	A1073T								2.065		
Hemopoietic and Lymphoid Tissue	C1053=					K1183R, P871L, S1436=, E1038G, S1634G, S694=, L771=					
Hemopoietic and Lymphoid Tissue	T221=									T243P	
Large Intestine	A1062V							675Dfs*6			
Large Intestine	A400V				2.119	D821Y		S3319Y, F1192C, D479Y, Y2997*			
Large Intestine	A50Lfs*3				2.189		2.357		2.134		
Large Intestine	A809=									E1010*	
Large Intestine	A878T									F776=	
Large Intestine	A879=									E1010*	
Large Intestine	C340R					D435Y				K95=	
Large Intestine	D125G							S562N		N280Tfs*8	
Large Intestine	D385N							P288S, R2896C, K817N, K981N,		E837K	

								D1177N, L2136V			
Large Intestine	D658G							K2446=		Y409H	
Large Intestine	D914N									R566H	
Large Intestine	G161=					S763F, K1732R, E577*		E2258K, S2052*, L29I, L414=, E1415=		T993=, E554K, R147=	
Large Intestine	G236R					E495=		K1565N			
Large Intestine	G30S					S763F, K1732R, E577*		E2258K, S2052*, L29I, L414=, E1415=		T993=, E554K, R147=	
Large Intestine	G326D			T297=							
Large Intestine	H202R					N976S, G401E		A1439=, T1505I		T733A, F440Lfs*12	
Large Intestine	H743Pfs*17							G995*			
Large Intestine	I1058=				2.094						
Large Intestine	I512S					K1254T		S3332Y, F701C, L951I, K956T			
Large Intestine	I989S					K1254T		S332Y, F701C, L951I, K956T			

Large Intestine	K56R				2.173	E572=		N337=		V221E	
Large Intestine	K984T				2.119	D821Y		S3319Y, F1192C, D479Y, Y2997*			
Large Intestine	L203=					G1100R		W1692Mfs*3, L1390Ffs*13			
Large Intestine	L611=							V2815=		P656=, K633=	
Large Intestine	N736=						2.37		2.45		
Large Intestine	P1068Rfs*46							S652=			
Large Intestine	P240=					E489G		E832G			-2.059
Large Intestine	P88=							P288S, R2896C, K817N, K981N, D1177N, L2136V		E837K	
Large Intestine	P88=					E572*		S1597Y, R2494Q, K722Q, I1047M, S2378A, S2670L, L3180R		L675R, S328Y	
Large Intestine	Q851*	F328=				S1524*, R7C					

Large Intestine	R1013C							D2661V, K3296N	2.571	D952G	2.212
Large Intestine	R1072H	S234L						K16R			
Large Intestine	R1072H			S305=				K16R			
Large Intestine	R200W			P336=				A248T			
Large Intestine	R223W		2.121								
Large Intestine	R655=					E495=		K1565N			
Large Intestine	R754Q	V222=						L1208*			
Large Intestine	R811Q	V222=						L1208*			
Large Intestine	S654N					I1318T, K690R, G57=		E97*, D281Y, S445Y, L613R, R645I, L901I, D1352Y, E2635G		P65=	
Large Intestine	T704Nfs*5					G552D					
Large Intestine	V1006I				2.992	S1434=					
Large Intestine	V1016=		2.168								
Large Intestine	V1025A							S652=			
Large Intestine	V1093I			S226F		E349V		E3096K, D3410=		R566H	
Large Intestine	V270D					E489G		E832G			-2.059
Large Intestine	V426M					E111Gfs*3					

Large Intestine	Y443=			S226F		E349V		E3096K, D3410=		R566H	
Liver	C418=				24.74						
Liver	I624V							G1376R, E2123K			
Liver	K173N					S590=					
Liver	K526N			Q221K				W2626L			
Liver	L68F		2.214								
Liver	P96=										2.043
Liver	R849=							G1376R, E2123K			
Liver	S1004=									A505=	
Liver	S322=									A505=	
Liver	V1012=			Y514C				V950F, C1365F, E2956*			
Liver	V627L			Q221K				W2626L			
Lung	A1062E							R2034H			
Lung	A50V		2.371				2.203		3.572		
Lung	C1053=							K1132=			
Lung	D790=									Q559R	
Lung	E669K									H1184N	
Lung	G677C					W1836C					
Lung	G815W		2.606								-2.443
Lung	P206=		2.629				4.953		3.812		2.533

Lung	Q184H					L52=		H3117N			
Lung	Q843*						3.818				4.126
Lung	R211S										-2.235
Lung	S713C						5.566	V2503F	3.936		2.575
Lung	T877=				9.084						
NS	I1023=					P1523L, E962K		P814=, L2587=			
NS	P279L							A248V			
NS	R811W							H2932Y			
Ovary	E995Q						7.874				
Ovary	I629F							S1230Lfs*9			-2.098
Ovary	Q215*					E111*					
Ovary	S691=							E425=, Q713L			
Ovary	V1011=							Q1934K			
Pancreas	R661Q							S2807=		E1010*, T696=	
Prostate	C463=	A195=	4.539			G1564=, S1180=	10.665	E2275D	10.58	A712V	
Prostate	H743=	A195=	4.539			G1564=, S1180=	10.665	E2275D	10.58	A712V	
Prostate	T1027=					P472H					
Skin	A400=							P2914Q			
Skin	D64N					G964V		E215G			
Skin	D698N									L9F	
Skin	D932Y					L1538I				E956*	
Skin	E296*							P110=			
Skin	E4K					T1706=		E1812*		H1126Q	

Skin	F1063=					Q202*					
Skin	F277=				3.324				2.058		
Skin	F303=					K608=, T333I					
Skin	F588=							L291=			
Skin	F817=						3.983				
Skin	G1017*							F1450=			
Skin	G187W						2.627				
Skin	G193R									P713L	
Skin	G236W						2.703	G1174C			
Skin	G391E				2.56						
Skin	G391R				2.56						
Skin	G677S					P633S					
Skin	G756C						2.486	P46H		T521=	-2.151
Skin	G781*						2.486	P46H		T521=	-2.151
Skin	G781R			L325F				V250M, S1985F		R365K	
Skin	G91C		3.827								
Skin	G921A							F3090=			
Skin	G928V					L1538I				E956*	
Skin	I1081=					L481=					
Skin	I719L	S182=				Q1299L, L269V		A1670=			
Skin	L287=					R1720L					
Skin	L372										2.64
Skin	L594F							E3152D			
Skin	L60F				4.605						
Skin	L920=								3.486	Q559*	
Skin	N89=					K608=, T333I					
Skin	P1068S							P46S		R510K	

Skin	P255S							P3282L			
Skin	P279S							Q2945*			
Skin	P355L					P875S					
Skin	P373H									S865L	
Skin	P724S						5.106				
Skin	P742H							P877Q, W3106L			2.892
Skin	Q184*							F701=			
Skin	Q533H								2.953		
Skin	Q533H	G308V									
Skin	Q701K				4.547						
Skin	R414Q					P633S					
Skin	S1067F					Q1800*		A1253=, G1696E			
Skin	S164L										5.933
Skin	S189=							E1876K			
Skin	S246L									P684L, S605F	
Skin	S564=		2.673					S2072=, P3320H			
Skin	T188=			P234T							
Skin	T521=					A1810V, P568F		P1702L		S582=	
Skin	T730=					A1810V, P568F		P1702L		S582=	
Skin	V943M					L1538I				E956*	
Skin	W294*					T1855I					
Skin	Y729N									M1?	
Small Intestine	I450F					I986Sfs*14		I605Nfs*11			

Soft Tissue	F614=						2.103		2.109		
Soft Tissue	V1078=		3.045								
Stomach	A670V							Q2539H			2.086
Stomach	C1053=							N372H			
Stomach	D385N						2.224	D946Rfs*13			
Stomach	G1010=				2.409						
Stomach	K530Q					S1501=, E1258G					
Stomach	L1002=					K1183R, P871L, S694=		N372H			
Stomach	L986S							T3033Lfs*29			
Stomach	Q473R					K1497R, T1246A, K1104E		T3033Lfs*29, P190H, L413V			
Stomach	Q473R							E3342			
Stomach	S423N							E3342			
Stomach	T813M	R171Q, D317N		T398A		G1366=		A1393V		S682P	
Stomach	V762A							N1619D, A2351T			
Stomach	Y387=						R18C				
Thyroid	A946V	E238D								K16R	
Thyroid	F915I					R691I					
Upper Aerodigestive	E334K					K1183R, P871L		N372H			

Upper Aerodigestive	L372F							R2784 W, L712=			
Upper Aerodigestive	P199S							P606L			
Upper Aerodigestive	P355L										2.248
Upper Aerodigestive	Q508*								5.116		2.424
Upper Aerodigestive	T61N					K1183R , P871L		N372H			
Upper Aerodigestive	V238L							T3033N fs*11			
Urinary Tract	D300N					L30F	2.404				
Urinary Tract	E374K						6.442		9.685		2.249
Urinary Tract	E668Q					Q1633 H					
Urinary Tract	F583=				2.201			T868I			
Urinary Tract	F79L		5.109				3.369		3.452		
Urinary Tract	G517S							Q1124*			
Urinary Tract	L416=					L30F	2.404				
Urinary Tract	L697=							V171=			
Urinary Tract	L92=					L30F					
Urinary Tract	S1067=		2.392				2.012		4.44		
Urinary Tract	T348=					Q667*					
